# Intramuscular administration of glyoxylate rescues swine from lethal cyanide poisoning and ameliorates the biochemical sequalae of cyanide intoxication

**DOI:** 10.1093/toxsci/kfac116

**Published:** 2022-11-03

**Authors:** Vik S Bebarta, Xu Shi, Shunning Zheng, Tara B Hendry-Hofer, Carter C Severance, Matthew M Behymer, Gerry R Boss, Sari Mahon, Matthew Brenner, Gregory T Knipp, Vincent Jo Davisson, Randall T Peterson, Calum A MacRae, Jared Rutter, Robert E Gerszten, Anjali K Nath

**Affiliations:** Department of Emergency Medicine, University of Colorado School of Medicine, Aurora, Colorado 80045, USA; Department of Cardiology, Beth Israel Deaconess Medical Center, Boston, Massachusetts 02115, USA; Department of Cardiology, Beth Israel Deaconess Medical Center, Boston, Massachusetts 02115, USA; Department of Emergency Medicine, University of Colorado School of Medicine, Aurora, Colorado 80045, USA; Department of Emergency Medicine, University of Colorado School of Medicine, Aurora, Colorado 80045, USA; Department of Industrial and Physical Pharmacy, Purdue University, West Lafayette, Indiana 47907, USA; Department of Medicine, University of California, San Diego, California 92093, USA; Department of Medicine, Beckman Laser Institute, University of California, Irvine, California 92697, USA; Department of Medicine, Beckman Laser Institute, University of California, Irvine, California 92697, USA; Department of Industrial and Physical Pharmacy, Purdue University, West Lafayette, Indiana 47907, USA; Department of Industrial and Physical Pharmacy, Purdue University, West Lafayette, Indiana 47907, USA; Department of Pharmacology and Toxicology, College of Pharmacy, University of Utah, Salt Lake City, Utah 84112, USA; Division of Cardiovascular Medicine, Brigham and Women’s Hospital, Boston, Massachusetts 02115, USA; Department of Biochemistry, Howard Hughes Medical Institute, University of Utah, Salt Lake City, Utah 84112, USA; Department of Cardiology, Beth Israel Deaconess Medical Center, Boston, Massachusetts 02115, USA; Broad Institute, Cambridge, Massachusetts 02142, USA; Harvard Medical School, Boston, Massachusetts 02115, USA; Department of Cardiology, Beth Israel Deaconess Medical Center, Boston, Massachusetts 02115, USA; Broad Institute, Cambridge, Massachusetts 02142, USA; Harvard Medical School, Boston, Massachusetts 02115, USA

**Keywords:** preclinical animal models, glyoxylate, medical countermeasures, cyanide antidotes, swine, metabolism, redox balance

## Abstract

Cyanide—a fast-acting poison—is easy to obtain given its widespread use in manufacturing industries. It is a high-threat chemical agent that poses a risk of occupational exposure in addition to being a terrorist agent. FDA-approved cyanide antidotes must be given intravenously, which is not practical in a mass casualty setting due to the time and skill required to obtain intravenous access. Glyoxylate is an endogenous metabolite that binds cyanide and reverses cyanide-induced redox imbalances independent of chelation. Efficacy and biochemical mechanistic studies in an FDA-approved preclinical animal model have not been reported. Therefore, in a swine model of cyanide poisoning, we evaluated the efficacy of intramuscular glyoxylate on clinical, metabolic, and biochemical endpoints. Animals were instrumented for continuous hemodynamic monitoring and infused with potassium cyanide. Following cyanide-induced apnea, saline control or glyoxylate was administered intramuscularly. Throughout the study, serial blood samples were collected for pharmacokinetic, metabolite, and biochemical studies, in addition, vital signs, hemodynamic parameters, and laboratory values were measured. Survival in glyoxylate-treated animals was 83% compared with 12% in saline-treated control animals (*p* < .01). Glyoxylate treatment improved physiological parameters including pulse oximetry, arterial oxygenation, respiration, and pH. In addition, levels of citric acid cycle metabolites returned to baseline levels by the end of the study. Moreover, glyoxylate exerted distinct effects on redox balance as compared with a cyanide-chelating countermeasure. In our preclinical swine model of lethal cyanide poisoning, intramuscular administration of the endogenous metabolite glyoxylate improved survival and clinical outcomes, and ameliorated the biochemical effects of cyanide.

Cyanide is a chemical reactant used in large quantities in industrial organic synthesis applications including nylon, fiber dyes, herbicides, polymers, nitriles, and other products (Hall *et al.*, 2015). The approximately 1.5 million tons of cyanide produced and transported within the United States, annually, elevates the risk of accidental or nefarious release (Cunningham, 2005; Dhara and Gassert, 2002; Dzombak *et al.*, 2005; Krakow, 2019). Acute effects of cyanide poisoning are cessation of breathing, termination of cellular respiration, and cardiovascular collapse, which rapidly leads to death (Kaita *et al.*, 2018). This poses occupational and civilian health risks as cyanide is a potent and fast-acting metabolic poison (Vogel et al., 1981).

The widespread use of cyanide in industrial settings, combined with its release during the combustion of cyanide-containing products, creates scenarios for a mass casualty incident resulting from fires, accidental spills, or terrorist attacks (Defense USAMRIoC, 2007; Dhara and Gassert, 2002; Eckstein and Maniscalco, 2006; Kales and Christiani, 2004). No antidotes exist against cyanide poisoning that first responders or bystanders can use to treat mass casualties in the prehospital setting. Existing FDA-approved cyanide antidotes must be given in large volumes intravenously over 15 min, followed by a potential second dose with an infusion rate ranging from 15 min to 2 h (CYANOKIT), both of which are not practical in a mass casualty setting due to the time, supplies, and skills required to start intravenous lines. Therefore, there is an unmet medical need for a fast-acting cyanide countermeasure that can be rapidly administered by intramuscular injection using an autoinjector (Jett and Yeung, 2010).

The cellular mechanism of cyanide toxicity is inhibition of complex IV of the electron transport chain, leading to blockade of aerobic respiration and disruption of metabolic pathways. The 2 FDA-approved antidotes for cyanide poisoning, Cyanokit (hydroxocobalamin) and Nithiodote (sodium nitrite and sodium thiosulfate), act by stoichiometric chelation or detoxification of cyanide (Borron and Baud, 2012). By leveraging metabolite profiling studies (Sips *et al.*, 2018) and high-throughput chemical screens in zebrafish (Nielson *et al.*, 2022), we identified a novel cyanide countermeasure that acts via multiple mechanisms to rescue cyanide-poisoned animals. We discovered that glyoxylate, an endogenous 2-carbon alpha-keto-acid, reacts with cyanide to form a cyanohydrin. Independent of chelation, glyoxylate reverses cyanide-induced redox imbalances. The underlying mechanism is not known. Notably, glyoxylate can be both oxidized and reduced—catalyzed by a handful of subcellular compartment-specific oxidoreductase enzymes—and these reactions are coupled to oxidation-reduction of NADH/NAD+ cofactors. Previously, in zebrafish, we found that lactate dehydrogenase (LDH) activity is required for glyoxylate-mediated protection against cyanide poisoning underscoring the cellular actions of glyoxylate-mediated rescue (Nielson *et al.*, 2022). This suggests that glyoxylate may also ameliorate the biological sequelae of cyanide intoxication.

Studies have been performed evaluating the efficacy of glyoxylate as a cyanide countermeasure in zebrafish and small mammalian models (Nielson *et al.*, 2022; Sips *et al.*, 2018). However, medical countermeasures for chemical toxicants must be FDA-approved under the Animal Rule using preclinical large-animal models (FDA, 2022; Jett and Yeung, 2010). Therefore, this study’s main objectives were to (1) assess glyoxylate’s efficacy in swine, an FDA-approved preclinical animal model that is favorable for human dosing because of the similarity to human anatomy, physiology, genome, and size (Swindle *et al.*, 2012), and (2) evaluate if the putative biochemical mechanisms of glyoxylate are conserved in large mammalian models. We used survival following exposure to a highly lethal cyanide dose as a primary outcome in addition to physiological and laboratory parameters, similar to those monitored during the care of critically ill patients, as secondary outcomes (Bebarta *et al.*, 2010; Fortin *et al.*, 2010; Hendry-Hofer *et al.*, 2020; Muncy *et al.*, 2012). Pharmacokinetic studies and metabolite profiling were also conducted to characterize the countermeasure biochemically. An additional objective was to evaluate the toxicity of acute glyoxylate exposure in rats. Together, these studies demonstrate that intramuscular administration of glyoxylate is rapidly absorbed, improves survival and physiological parameters, induces distinct effects on redox balance as compared with a cyanide-chelating agent, and restores metabolic profiles in cyanide-poisoned swine. Finally, glyoxylate was well tolerated in rats at 1- and 5-days post-administration. In summary, these results reveal a path toward developing a novel class of cyanide countermeasures that are based on the endogenous metabolite glyoxylate.

## Materials and methods

### Swine cyanide model

**Figure 1.**
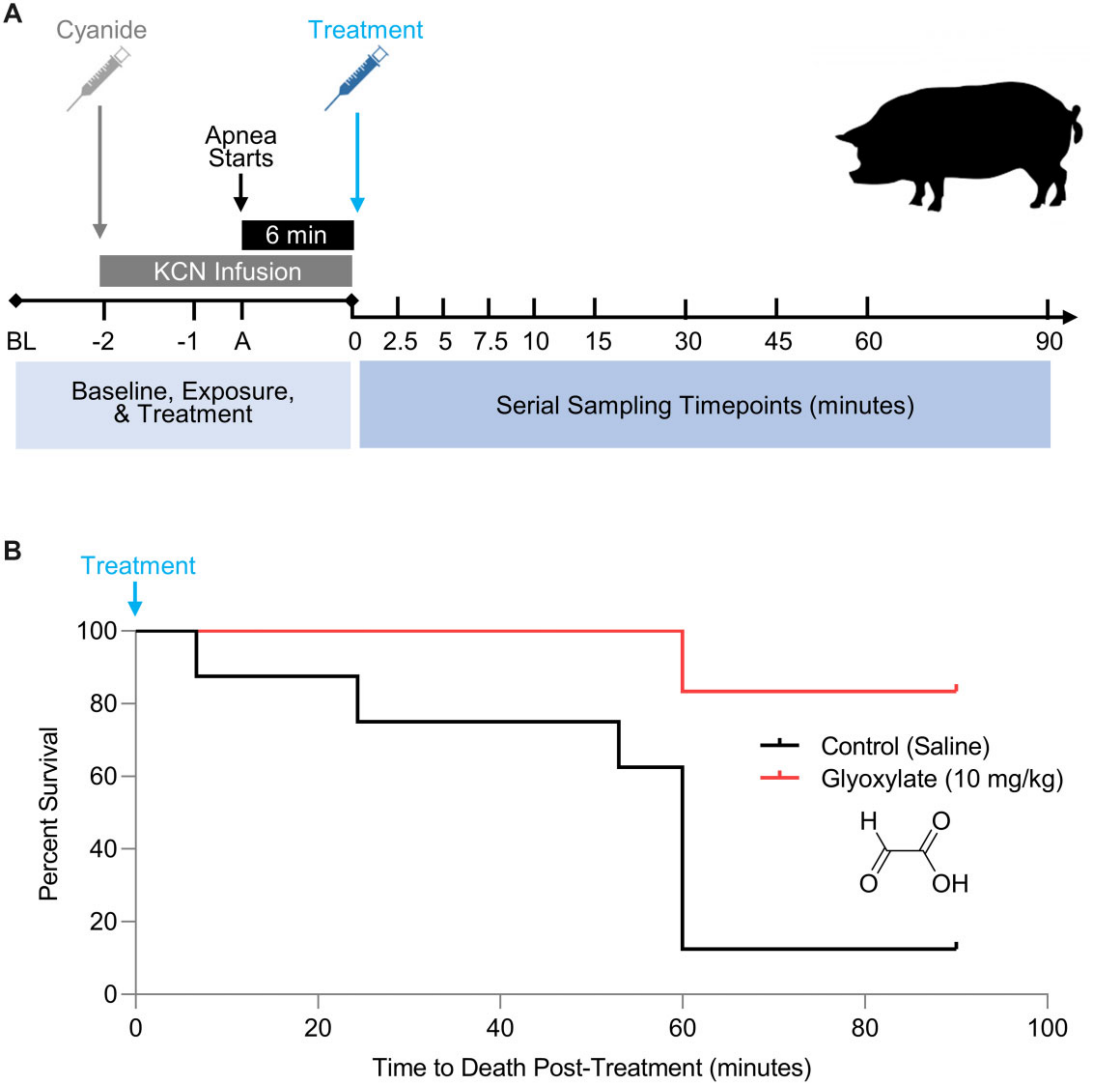
Intramuscular administration of glyoxylate improves survival in cyanide-poisoned animals. A, Overview of the experimental procedure in the swine cyanide model. We employed a physiological trigger-to-treatment model. In our model, cyanide was infused (gray bar) and continued for a period of 6 min after the start of apnea (black bar)—6 min of apnea was the trigger to inject treatment (blue arrow), and following treatment, the cyanide infusion was stopped. Definition of discontinuous time points: BL, baseline sample/measurement; −2, sample/measurement was taken immediately after starting the cyanide infusion; −1, sample/measurement was taken 5 min post-cyanide infusion; A, sample/measurement was taken 30 s after the start of apnea (defined as ≤6 breaths per minute for 20 s as determined by capnography); 0, after 6 min of apnea, the treatment was injected, and the sample was taken immediately after the injection of treatment. B, Kaplan‐Meier plot of survival in control (*n* = 8; black line) versus glyoxylate (*n* = 6; red line) treated animals (*p* < .001).

We use approximately 50 kg Yorkshire swine in our model. Their large size approximates that of a human, making scaling drug doses to a human dose relatively straightforward. Moreover, their respiration rate, physiology of ventilatory responses, and circulatory system are similar to humans which makes this a good preclinical model. Finally, in the swine model it is feasible to capture multiple clinically relevant endpoints including hemodynamic, physiological, metabolic, and laboratory parameters that can be used to assess outcomes. Our exposure paradigm is shown in Figure 1A. This paradigm evaluates the antidotal efficacy of compounds in apneic animals that were exposed to a lethal dose of cyanide. We used a non-ventilated cyanide infusion model. Because the pKa of HCN is 9.2, cyanide exists almost exclusively as HCN at physiological pH, and infusing a cyanide salt generates HCN, the form of cyanide absorbed from the lungs or stomach. Thus, a cyanide infusion model yields the same end product as an inhalation or ingestion model, but has the advantage of knowing the exact amount of cyanide the animal receives. In our model, we employed a symptomatic trigger-to-treatment model; 6 min of apnea (Figure 1A, black bar) was the trigger to inject treatment (Figure 1A, blue arrow). Pigs are held at apnea for 6 min and subsequently administered with saline or antidote. Invasive blood pressure is monitored continuously and blood sampling occurs serially throughout both the exposure and recovery phases of the model (Figure 1A).

Female Yorkshire swine (*Sus domesticus*) (Oak Hill Genetics) weighing 45–55 kg were used for this study. Anesthesia was induced with intramuscular administration of 10–20 mg/kg ketamine and nosecone isoflurane. Animals were intubated with an 8.0 mm cuffed endotracheal tube and peripheral venous access is obtained. Sedation was maintained using the Drager Apollo anesthesia machine with 1%–3% isoflurane. If needed to maintain an end-tidal CO_2_ of 35–45 mmHg, pressure support ventilation was provided using a driving pressure of 5–8 cmH_2_O, a peak expiratory pressure of 5 cmH_2_O, and 0.4 FiO_2_. Before central line placement, a 7.5 -ml/kg bolus of 0.9% saline was given. The external jugular vein and femoral artery were visualized using the M9 ultrasound system (Mindray) and central venous and arterial access is obtained. The Drager Infinity Delta Monitor recorded pulse oximetry, body temperature, invasive blood pressure, and ECG throughout the experiment. Invasive hemodynamic variables were measured via pulmonary artery catheterization using an 8‐French Swan Ganz CCOmbo catheter and the Edwards Vigilance II monitor. Arterial blood was obtained at baseline, at 30 s post-apnea, and at the indicated times and analyzed with a handheld blood analyzer (iSTAT, Abbott); parameters measured included paO_2_, paCO_2_, lactate, and pH. Before experimentation mechanical ventilation, if required, was weaned, allowing the animal to breathe spontaneously. Isoflurane, as well as FiO_2_, was also weaned to 0.8%–1.5% and 0.21, respectively. The control animals were treated with saline in place of the antidote. These controls were run periodically throughout the year to ensure negligible cohort-to-cohort variation. Sodium glyoxylate monohydrate (Sigma Aldrich, catalogue no. G10601) was dissolved in 0.1 M phosphate buffer (Ca^2+^ and Mg^2+^‐free) at 140 mg/ml and at final formulation pH 7.5. We calculated that at a dose of 10 mg/kg glyoxylate, a 50-kg pig in the treatment group received a total of 101 mg of sodium (sodium in sodium glyoxylate + sodium in the diluent) versus a control pig that received 12.8 mg sodium (saline injection). This is a small amount of sodium as compared with the sodium in the bolus of 0.9% saline required during central line placement.

Potassium cyanide (Sigma Aldrich) diluted in saline was delivered via continuous infusion into the right jugular vein (0.17 mg/kg/min; average total KCN dose of 88.5 ± 10.2 mg: using allometric scaling from swine to humans, the dose of cyanide delivered to swine would scale to a human exposure of 1.9 mg/kg of potassium cyanide). Cyanide infusion was continued until 6 min after apnea occurs, defined as ≤6 breaths per minute for 20 s as determined by capnography. At this timepoint, animals are treated with either glyoxylate (10 mg/kg) or saline (control) injected into the right vastus medialis muscle in a volume of approximately 3.5 ml, and the cyanide infusion is terminated (Figure 1A). Following the treatment, animals were observed continuously for 90 min or until death, defined as a mean arterial pressure of less than 30 mmHg for 10 min, or sustained apnea (less than 6 breaths per minute for 30 min), or pulse oximetry ≤70% for 30 consecutive minutes, confirmed by arterial blood oxygenation of ≤50 mmHg. The antidotal efficacy of glyoxylate was evaluated by assessing the primary outcome, survival at 90 min post-treatment, in addition to secondary outcomes (clinical, laboratory, and metabolic parameters). At the end of the study, all animals were euthanized with an intravenous administration of 100 mg/kg sodium pentobarbital. All methods were carried out in accordance with the regulations and guidelines of the Animal Welfare Act and the American Association for Accreditation of Laboratory Animal Care. The IACUC committee at the University of Colorado approved all experimental protocols.

### Targeted metabolomics

Plasma samples (30 µl) were deproteinized using 70 µl of acetonitrile/methanol (75:25; v/v) containing stable isotope-labeled and deuterated internal standards 25 μM thymine-d_4_, 10 μM inosine-^15^N_4_, 10 μM citrulline-d_7_, 25 μM phenylalanine-d_8_, and 10 μM valine-d_8_. Samples were vortexed briefly and then centrifuged (10 000 rpm, 10 min). Supernatants were transferred to glass autosampler vials containing glass inserts and subjected to LC-MS/MS analysis. The samples were separated using a 2.1 × 100 mm 3.5-μm Xbridge amide column (Waters). Mobile phase A was 95:5 (v/v) water/acetonitrile, with 20 mM ammonium acetate and 20 mM ammonium hydroxide (pH 9.5). Mobile phase B was acetonitrile. For amide-negative mode, the chromatography system consisted of a 1260 Infinity autosampler (Agilent) connected to a 1290 Infinity HPLC binary pump system (Agilent). The eluents were detected in negative mode on a coupled 6490 QQQ mass spectrometry equipped with an electrospray ionization source. The settings were as follows: sheath gas temperature, 400°C; sheath gas flow, 12 l/min; drying gas temperature, 290°C; drying gas flow, 15 l/min; capillary, 4000 V; nozzle pressure, 30 psi; nozzle voltage, 500 V; and delta EMV, 200 V (Kimberly *et al.*, 2017; Morningstar *et al.*, 2019). LC‐MS data were quantified using Agilent MassHunter Quantitative Analysis software. All metabolite peaks were manually reviewed for peak quality in a blinded manner and compared against a known standard to confirm identity. Pooled plasma was interspersed throughout the run at regular intervals to monitor the temporal drift in mass spectrometry performance and assess the variability of each metabolite. See Supplementary Methods and Supplementary Table 1 for additional details.

### Pharmacokinetics of glyoxylate and oxalate

Baseline and serial blood sampling were performed over 110 min and analyzed using quantitative LC-MS/MS analysis. Multiple reaction monitoring transitions were optimized for glyoxylate and oxalate on our Amide platform (Nielson *et al.*, 2022). Following optimization, calibration standards were run to generate a standard curve for each species. Glyoxylate-^13^C_2_ standard curve was generated in pooled reference plasma using isotopically labeled glyoxylate. The standard curve was linear between 122 nM and 500 μM. To generate the standard curve for oxalate, we spiked pooled reference plasma with oxalate and made serial dilutions that were analyzed by LC-MS/MS. The standard curve was linear between 61 nM and 125 μM. Next, experimental samples were run and signals for glyoxylate and oxalate were compared with the calibration curve in order to determine their absolute concentration.

### Rat toxicity study

Male (*n* = 3) and female (*n* = 3) Sprague Dawley rats (Envigo and Inotiv) with weight ranges of 225–250 g were used for these studies which were performed at the Purdue Translational Pharmacology and Clinical Veterinary Pathology Laboratories. Sodium glyoxylate was prepared in 0.1 M phosphate buffer (Ca^2+^ and Mg^2+^‐free) at a final pH 7.5. Glyoxylate (60 and 145 mg/kg) was administered by IP injection in approximately 150 µl of solution adjusting for the weight of individual animal. Similarly, control animals received 150 µl of vehicle adjusting for weight of individual animals. For dosing at 145 mg/kg, the formulation was adjusted for ionic strength and diluted with purified water and delivered by injecting approximately 1.10 ml of solution adjusting for weight of each individual animal. Blood was drawn at 1- and 5-days post-injection and then processed for Comprehensive Metabolite Panel and Complete Blood Count Panel. References values were obtained from Envigo and Inotiv, and the Purdue Translational Pharmacology Core. At the end of the study, the animals were euthanized following the PHS Policy on the Human Care and Use of Animals, Guide for the Use and Care of Laboratory Animals. All methods were carried out in accordance with the regulations and guidelines of the Animal Welfare Act and the American Association for Accreditation of Laboratory Animal Care. The IACUC committee at Purdue University approved all experimental protocols.

### Statistics

The log-rank test (Mantel-Cox) was used to compare the survival distributions of glyoxylate-treated versus vehicle-treated swine in the lethal cyanide model. Physiological characteristics and laboratory values were analyzed using Welch’s *t* tests. For the metabolite data, we normalized metabolite levels in animals treated with cyanide to the value of their baseline (before cyanide infusion) peak area on a metabolite-by-metabolite basis, and then calculated the mean and standard deviation. Significance was assessed using 2-tailed non-paired Student *t* tests to compare the peak areas of baseline (before cyanide infusion) samples to end of the cyanide infusion and end of study samples. For the rat toxicity testing, statistical analysis was performed using a 2-way ANOVA with stacked repeated measures and using the Šídák’s multiple comparisons test to compare the means of treated animals’ versus vehicle for each value in the complete blood count and comprehensive metabolite panels. For the rat toxicity studies, we used an *n* = 6 (pooled: 3 males and 3 females); these studies are powered for pooled gender effectiveness (power of ≥ 80% with 95% CI). We did not observe gender effects, therefore did not repeat the studies in another 3 male and 3 female rats to achieve sufficient power for gender effects. For the swine model, the sample size was determined by a *χ*^2^ test, with an *α*  =  0.05 and power = 0.80; from pilot studies and previous work, we expected 90% lethality in untreated pigs and assumed 85% survival in the treatment group, thus a sample size of 8 was used for untreated pigs and 6 for treated pigs. For metabolite profiling, based on our previous experience profiling citric acid cycle metabolites in the swine model, a minimum *n* = 3 is required to be sufficiently powered when both baseline and serial samples are available in the same animal. In this study, we leveraged all available samples collected from the efficacy study and used a sample size of 3–7 animals per group for mass spectrometry studies. For the 10 anaerobic and aerobic metabolites assessed for return to baseline (Table 4), to adjust for multiple hypothesis testing, we applied a 2-stage step-up method (Benjamini, Krieger, and Yekutieli) for false discovery rate (FDR) adjustment with a threshold for statistical significance of *q *<* *0.05.

**Table 4.** Metabolite values at death or end of study

Metabolite	Control (*n*= 7)	Glyoxylate (*n*= 6)	*p* value	FDR *q*
Glucose	0.76 ± 0.22	1.02 ± 0.19	.0541	0.0681
Pyruvate	2.86 ± 0.87	2.72 ± 1.28	.8285	0.6013
Lactate	3.58 ± 1.33	2.32 ± 1.76	.1949	0.3619
Oxaloacetate	1.76 ± 0.61	1.19 ± 0.23	.0745	0.0681
Citrate/isocitrate	0.99 ± 0.09	1.19 ± 0.21	.0700	0.0604
Aconitate	1.28 ± 0.17	1.23 ± 0.13	.6090	0.4808
Alpha-ketoglutarate	3.27 ± 1.32	1.77 ± 0.70	.0410	0.0604
Succinate	34.7 ± 30.9	2.21 ± 1.9	.0497	0.0212
Fumarate	18.9 ± 11.6	3.12 ± 2.19	.0203	0.0159
Malate	4.46 ± 1.31	1.95 ± 0.93	.0041	0.0159

Data were calculated from the integrated peak area (counts) using mass spectrometry software, and presented as fold change normalized to baseline and are the mean ± standard deviation. To adjust for multiple hypothesis testing, we applied a 2-stage step-up method (Benjamini, Krieger, and Yekutieli) for FDR adjustment with a threshold for statistical significance of *q *<* *0.05.

## Results

### Baseline characteristics of study subjects

At baseline, swine in both groups (vehicle control and glyoxylate treatment) exhibited similar weight, lactate, pH, mean arterial blood pressure (MAP), pulse oximetry, arterial blood oxygenation, respiratory rate, and minute volume (Table 1). In addition, at apnea (defined as ≤6 breaths per minute for 20 s as determined by capnography) and before treatment was administered, no significant differences in physiological parameters, respiratory parameters, and laboratory values were observed between groups (Table 2). These findings indicate a similar magnitude in cyanide-induced toxicities in both experimental and control groups; for example, both groups exhibited an equivalent amount of apnea-induced reduction in arterial blood oxygenation (control = 34.6 ± 7.78 versus treatment = 31.8 ± 4.07 mmHg; Table 2, *p* = n.s.).

**Table 1.** Baseline physiological characteristics of swine in the control and treatment groups

	Control (*n*= 8)	Glyoxylate (*n*= 6)	Difference in means	95% CI difference^*a*^
Weight (kg)	48.4 ± 4.40	46.6 ± 1.60	−1.83 ± 1.69	−5.63 to 1.97
Lactate (mmol/l)	1.02 ± 0.52	0.90 ± 0.35	−0.13 ± 0.23	−0.64 to 0.39
pH	7.41 ± 0.03	7.41 ± 0.06	−0.003 ± 0.03	−0.07 to 0.06
MAP (mmHg)	78.9 ± 3.36	70.3 ± 8.96	−8.54 ± 3.85	−17.93 to 0.845
Arterial blood oxygenation (mmHg)	67.5 ± 9.09	64.3 ± 5.35	−3.17 ± 3.89	−11.67 to 5.34
Pulse oximetry (percent oxygen saturation)	92.4 ± 2.97	93.5 ± 2.88	1.13 ± 1.58	−2.34 to 4.59
RR (breaths per min)	29.25 ± 8.36	29.83 ± 9.48	0.58 ± 4.87	−10.25 to 11.42
Minute volume (l per min)	7.08 ± 1.3	6.77 ± 1.27	−0.31 ± 0.69	−1.83 to 1.22

kg, kilograms; mmol/l, millimoles per liter; mmHg, millimeters of mercury; MAP, mean arterial blood pressure; RR, respiratory rate.

Data presented as mean ± standard deviation.

a There were no significant differences in laboratory values, hemodynamics, or respiratory rate at baseline.

**Table 2.** Laboratory values and hemodynamic characteristics of swine in the control and treatment groups before treatment^*a*^

	Control (*n*= 8)	Glyoxylate (*n*= 6)	Difference in means	95% CI difference^*c*^
Lactate (mmol/l)	2.05 ± 0.88	1.70 ± 0.77	−0.35 ± 0.44	−1.31 to 0.616
pH	7.38 ± 0.05	7.39 ± 0.05	0.003 ± 0.027	−0.055 to 0.061
MAP (mmHg)	72.0 ± 15.9	65.9 ± 10.4	−6.14 ± 7.19	−22.1 to 9.79
Arterial blood oxygenation (mmHg)	34.6 ± 7.78	31.8 ± 4.07	−2.79 ± 3.21	−9.87 to 4.28
Pulse oximetry (percent oxygen saturation)**^*b*^**	86.9 ± 11.4	91.8 ± 6.01	4.96 ± 4.73	−5.45 to 15.4
RR (breaths per min)	3.57 ± 0.94	3.17 ± 0.75	−0.41 ± 0.40	−1.27 to 0.46

mmol/l, millimoles per liter; mmHg, millimeters of mercury; MAP, mean arterial blood pressure; RR, respiratory rate.

Data presented as mean ± standard deviation.

a Measurements were taken 30 s after the start of apnea (defined as ≤6 breaths per minute for 20 s as determined by capnography) and before treatment was administered.

b Due to technological differences, there is a time delay for pulse oximetry to reflect a decrease in oxygenation as compared with arterial blood oxygenation measurements.

c There were no significant differences in laboratory values, hemodynamics, or respiratory rate at treatment.

### Survival following high dose cyanide exposure in control and treatment groups

To determine the antidotal efficacy of glyoxylate against cyanide poisoning, nonventilated pigs were anesthetized and infused with KCN (0.17 mg/kg/min IV) until 6 min beyond the onset of cyanide-induced apnea. At this timepoint, saline or glyoxylate was injected intramuscularly (IM) and the cyanide infusion was terminated (Figure 1A). Establishing an efficacious dose for glyoxylate used allometric scaling from rabbit (Nielson *et al.*, 2022) to estimate a starting dose of 25 mg/kg. Initial dose refinement was conducted in 7 pigs and covered a dose range of 8–25 mg/kg.

Survival was assessed at 90 min, where the primary outcome showed 83% alive in the treatment group (10 mg/kg glyoxylate, *n* = 6) compared with 12% in the vehicle control group (saline, *n* = 8) (Figure 1B;*p* = .01). In the control group, 7 of the 8 animals died by 60 min post-cyanide infusion with a mean survival time of 51.7 ± 25.4 min as compared with glyoxylate-treated animals with a mean survival time of 85.0 ± 13.4 min (*p* = .008). Kaplan-Meier survival analysis indicated a significant difference in survival between the control and treatment groups (log-rank *p* = .01; Figure 1B).

### Glyoxylate treatment improves clinical parameters and laboratory values in cyanide-poisoned animals

**Figure 2.**
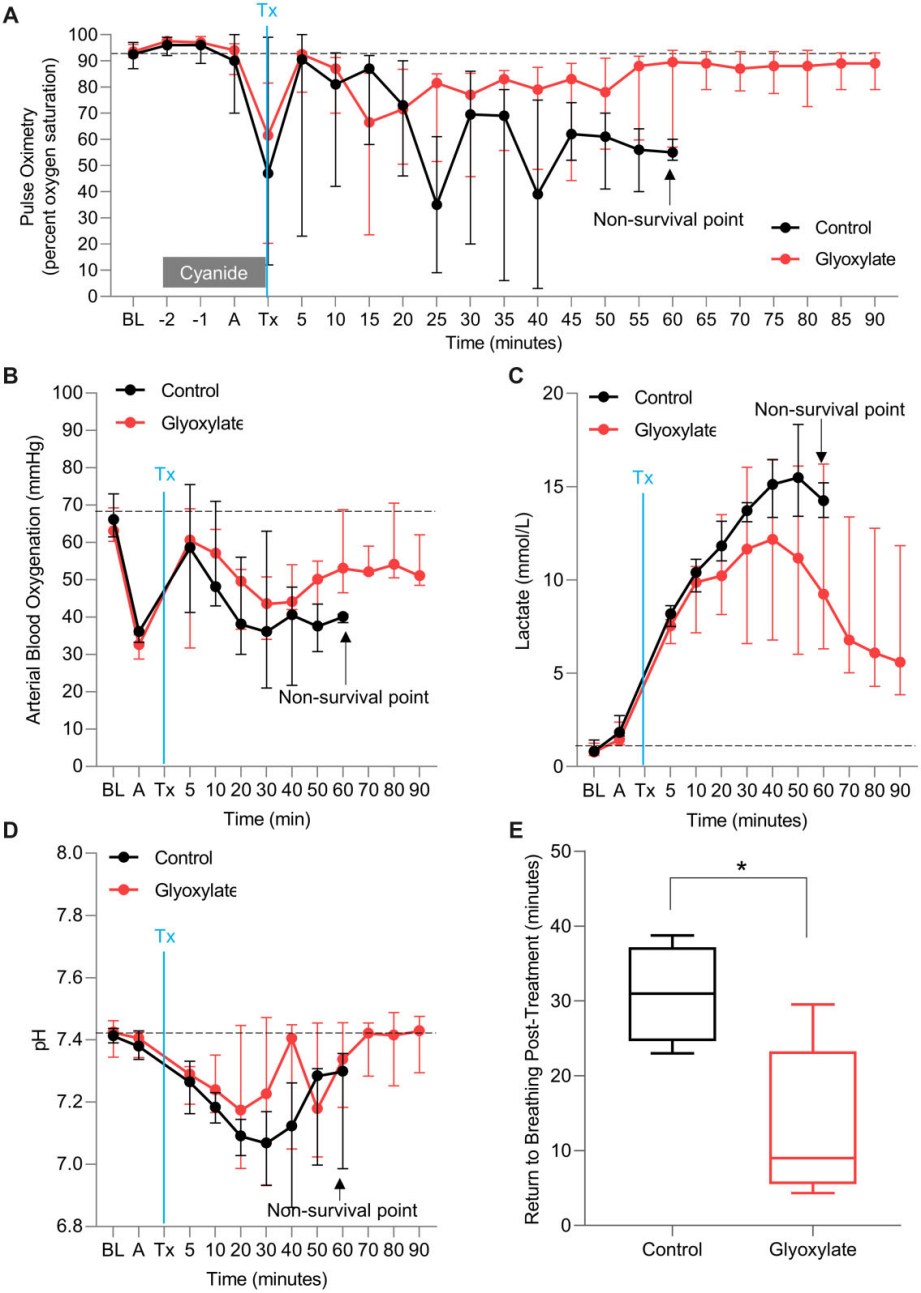
Glyoxylate treatment improves clinical parameters and laboratory values in cyanide-poisoned animals. (A) Pulse oximetry (a surrogate marker for tissue oxygenation), (B) arterial blood oxygenation (a direct measure of oxygen tension in arterial blood, ie, lung function), (C) lactate concentration, and (D) pH value of cyanide-poisoned swine administered vehicle control (black line) or 10 mg/kg glyoxylate (red line); note: in the glyoxylate group, pH data is skewed downwards at time point 50 due the inclusion of data from an animal that died at time point 60. The dashed line represents the baseline of each measurement before the treatment. BL, baseline measurement; −2, measurement was taken immediately after starting the cyanide infusion; −1, measurement was taken 5 min post-cyanide infusion; A, measurement was taken 30 s after the start of apnea (defined as ≤6 breaths per minute for 20 s as determined by capnography); Tx, after 6 min of apnea, the treatment was injected, and the sample was taken immediately after the injection of treatment (note: Tx timepoint was not collected for arterial blood oxygenation, lactate, and pH). Data presented as median with the interquartile range. See Table 3 for statistics. E, Box and whisker plot of time to return to breathing, defined as a respiration rate of ≥ 7 breaths per minute, post-treatment with vehicle control or glyoxylate treatment.

Pulse oximetry, arterial blood oxygenation, lactate, pH, and respiration were continuously measured for the duration of the study to monitor cyanide-induced hypoxia (Figs. 2A and 2B), hyperlactatemia (Figure 2C), acidemia (Figure 2D), and apnea (Figure 2E). Following cyanide-induced apnea, 100% of glyoxylate-treated animals (*n* = 6) returned to breathing, defined as a respiration rate of ≥ 7 breaths per minute. By contrast, only 50% of control-treated animals returned to breathing (*n* = 4) and the other 50% remained apneic until death (*n* = 4). In the animals that returned to breathing, the time to return to breathing was significantly improved in glyoxylate-treated animals as compared with control animals (13.32 ± 10.23 vs 30.91 ± 6.54 min, *p* = .0173; mean difference: −17.59 ± 5.62 min, 95% CI: −28.29 to −6.88, Figure 2E). Cyanide infusion induced hypoxia, as evidenced by decreased pulse oximetry and arterial blood oxygenation (Figs. 2A and 2B). Animals treated with glyoxylate exhibited significantly improved pulse oximetry (78.2 ± 21.7 vs 39.5 ± 28.3; *p* = .0135) and arterial blood oxygenation (52.8 ± 8.6 vs 39.1 ± 7.0; *p* = .0105) as compared with vehicle controls (Table 3 and Figs. 2A and 2B). In both glyoxylate-treated and vehicle-treated animals, lactate remained elevated at the end of study, 8.8 ± 5.8 mmol/l versus 14.0 ± 3.8 mmol/l (Figure 2C and Table 3; *p* = n.s.). Finally, pH returned to 7.4 at *t* = 40 min post-glyoxylate treatment while control animals remained acidotic until death (Figure 2D). In sum, these clinical and laboratory parameters recovery indicates that glyoxylate treatment improves recovery of physiological homeostasis in cyanide-poisoned animals.

**Table 3.** Animal characteristics at death or end of study

	Control (*n*= 8)	Glyoxylate (*n*= 6)	Difference in means	95% CI difference	*p* value
Lactate (mmol/l)	14.0 ± 3.8	8.8 ± 5.8	−5.2 ± 2.7	−11.4 to 1.02	.0904
pH	7.15 ± 0.21	7.34 ± 0.16	0.19 ± 0.10	−0.03 to 0.40	.0816
MAP (mmHg)	60.3 ± 29.0	72.8 ± 10.3	12.6 ± 11.1	−12.4 to 37.6	.2856
Arterial blood oxygenation (mmHg)	39.1 ± 7.0	52.8 ± 8.6	13.7 ± 4.3	4.0 to 23.4	.0105
Pulse oximetry (percent oxygen saturation)	39.5 ± 28.3	78.2 ± 21.7	38.7 ± 13.4	9.5 to 67.8	.0135

mmol/l, millimoles per liter; mmHg, millimeters of mercury; MAP, mean arterial blood pressure.

Data presented as mean ± standard deviation.

### Recovery of citric acid cycle metabolism in cyanide-poisoned animals treated with glyoxylate

**Figure 3.**
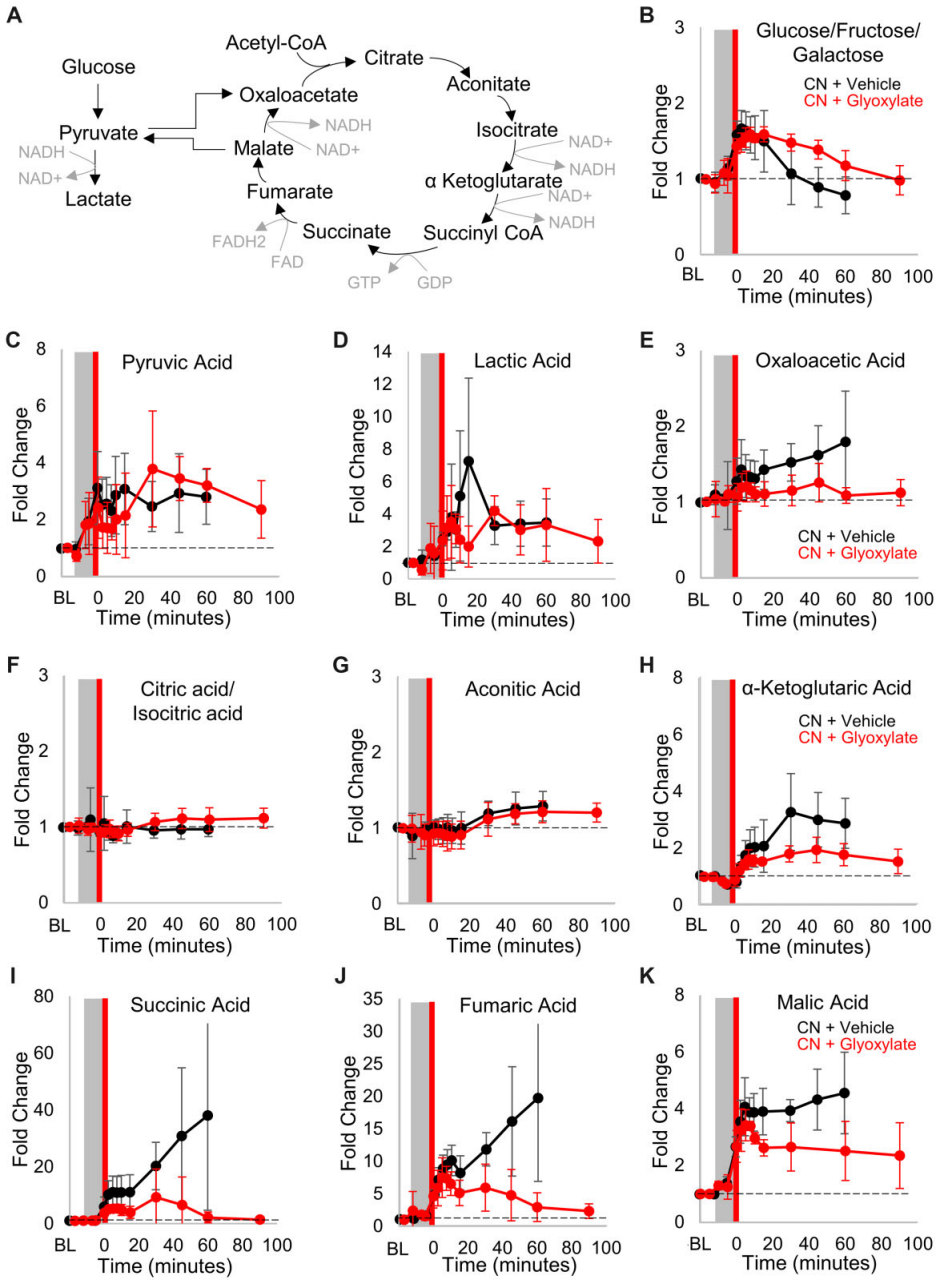
Recovery of citric acid cycle metabolism in cyanide-poisoned animals treated with glyoxylate. A, Diagram of aerobic (glycolysis) and anaerobic (citric acid cycle) metabolism. Plasma levels of (B) glucose/fructose/galactose, (C) pyruvic acid, (D) lactic acid, (E) oxaloacetic acid, (F) citric acid/isocitric acid, (G) aconitic acid, (H) α‐ketoglutaric acid, (I) succinic acid, (J) fumaric acid, and (K) malic acid in swine treated with cyanide (gray box) and subsequently, at *t* = 0, administered vehicle control (black circles; *n* = 7) or 10 mg/kg glyoxylate IM (red circles; *n* = 5–6). The dashed line represents the baseline of each measurement. Data were normalized to baseline and presented as mean ± standard deviation. See Table 4 for statistics.

Previously, we demonstrated that cyanide poisoning induces substantially increased second-span citric acid cycle metabolites (Morningstar *et al.*, 2019; Nielson *et al.*, 2022; Sips *et al.*, 2018). Moreover, efficacious cyanide countermeasures restored normal levels of these metabolites. Thus, an additional secondary outcome in our study was the recovery of citric acid cycle metabolism. Baseline and serial blood sampling were performed over 110 min (Figure 1A) and subsequently plasma metabolites were measured using LC-MS/MS (Figs. 3A–K). Metabolite differences were compared between baseline and endpoint samples to evaluate amelioration and a trend toward normalization by the experimental endpoint.

In glyoxylate-treated swine, plasma levels of alpha-ketoglutarate, succinate, fumarate, and malate significantly increased reaching peak fold change of 1.9 ± 0.4 (*t* = 45), 9.3 ± 9.9 (*t* = 30), 7.4 ± 1.8 (*t* = 7.5), 3.1 ± 0.5 (*t* = 5), respectively (Figs. 3H–K; *p* = .0007, .0001, n.s., .0004, respectively; all FDR ≤ 0.05). All 4 metabolites returned to baseline by *t* = 90 (*p* = n.s.). By contrast, first-span metabolites (oxaloacetate, citrate, and aconitate) did not change significantly during the study (Figs. 3E–G). In comparison with cyanide-poisoned animals that received the vehicle control, glyoxylate administration significantly improved fumarate (*p* = .0203), succinate (*p* = .0497), and malate (*p* = .0041) at death or the end of the study comparative to the controls (Table 4 and Figs. 3H–K; all FDR ≤ 0.05). Lactate and pyruvate remained elevated at death or the end of the study (Figs. 3C and 3D). However, notably, between 5 and 15 min post-treatment lactate rapidly decreased in glyoxylate-treated animals by −42% whereas it continued to rapidly rise by +89% in cyanide-poisoned animals that received saline instead of an antidote (Figure 3D). This raises the possibility that a burst in citric acid cycle activity between the *t* = 5 and *t* = 15 timepoints contributes to the beneficial effects of glyoxylate on clinical outcomes and survival. Concordantly, during the same timeframe second-span metabolites were consumed and, subsequently, continued to decline toward baseline in glyoxylate-treated animals as compared with controls which exhibited continued increase in these metabolites (Figs. 3H–K). This suggests a shift from glycolysis to activation of citric acid cycle metabolism in glyoxylate-treated animals.

### Pharmacokinetic profile of glyoxylate following intramuscular administration in swine

**Figure 4.**
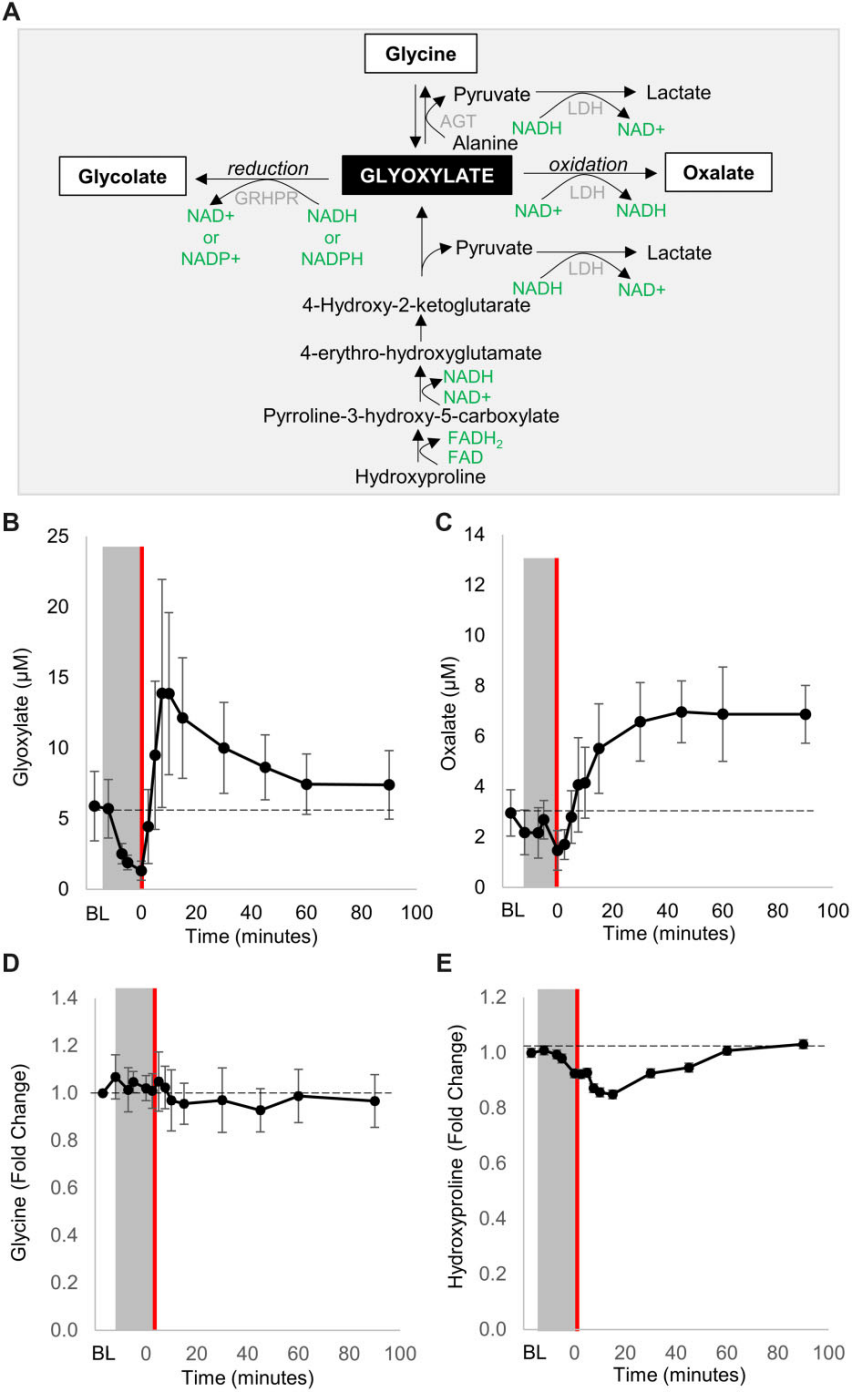
Pharmacokinetic profile of glyoxylate following intramuscular administration in swine. A, Diagram of the metabolic fates of glyoxylate. Pharmacokinetic profile for (B) glyoxylate and (C) oxalate, and fold change of (D) glycine, and (E) hydroxyproline in the plasma of swine exposed to cyanide (gray box) followed by intramuscular administration of 10 mg/kg glyoxylate at *t* = 0. The dashed line represents the baseline of each measurement. Data are presented as mean ± standard deviation (*n* = 5–6). LDH, lactate dehydrogenase; GRHPR, glyoxylate reductase/hydroxypyruvate reductase; NAD+/NADH, nicotinamide adenine dinucleotide; NADP+/NADPH, nicotinamide adenine dinucleotide phosphate; and FAD/FADH_2_, flavin adenine dinucleotide.

In mammals, the major pathways of glyoxylate metabolism include the formation of oxalate, glycolate, and glycine (Figure 4A). At the start of the study, baseline concentration of glyoxylate level was 5.90 ± 2.46 µM. At the end of the approximately 12-min cyanide infusion, glyoxylate concentration decreased by 4.5-fold to 1.31 ± 0.68 µM (Figure 4B;*p* = .005). Following IM injection of 10 mg/kg glyoxylate (*n* = 6), plasma concentration of glyoxylate peaked at 9.5 ± 5.4 min (*C*_max_ = 16.2 ± 6.1 µM; Supplementary Table 2), a 12.4-fold increase from time of treatment, that is, *t* = 0 (*p* = .005). At 60 min post-injection, glyoxylate concentration returned to near baseline concentration (7.40 ± 2.43 µM; *p* = n.s.). These data demonstrate that glyoxylate is very rapidly absorbed from the injection site and rapidly cleared from circulation.

Next, we quantified the glyoxylate-derived metabolite, oxalate, and found substantial increases. At the start of the study, baseline concentration of oxalate level was 2.96 ± 0.92 µM. At the end of cyanide infusion, oxalate concentration decreased by 2.0-fold to 1.47 ± 0.79 µM (Figure 4C;*p* = .04). Following IM injection of glyoxylate, plasma concentration of oxalate steadily increased and plateaued at 45 min post-injection reaching *C*_max_ of 8.0 ± 0.5 µM (*T*_max_ = 65.0 ± 22.6 min; Supplementary Table 2), a 5.5-fold increase from time of treatment (Figure 4C;*p* = 4E-08). In addition to oxidation of glyoxylate to oxalate, reduction of glyoxylate produces glycolate (Figure 4A); however, our platform does not measure that metabolite. Lastly, glycine levels did not change (Figure 4D) and hydroxyproline—the mitochondrial source of glyoxylate and a collagen degradation product (Jiang *et al.*, 2012; Knight and Holmes, 2005)—decreased by 16% (*p* = .003) which may relate to the observed decrease in endogenous glyoxylate during cyanide infusion (Figure 4E).

### Plasma biomarkers that reflect intracellular redox balance improve in glyoxylate-treated animals

Glyoxylate has a handful of possible metabolic fates (Figure 4A). However, its metabolite products (glycine, oxalate, and glycolate) provide no protection against cyanide poisoning (Nielson *et al.*, 2022). Importantly, the oxidation/reduction of glyoxylate is coupled to the oxidation/reduction of the cofactors NADH/NAD+ and NADPH/NADP+ (Figure 4A and Supplementary Figure 2). Therefore, the rapid absorption of glyoxylate and its subsequent metabolism may modify the imbalances in essential cofactors caused by cyanide intoxication.

**Figure 5.**
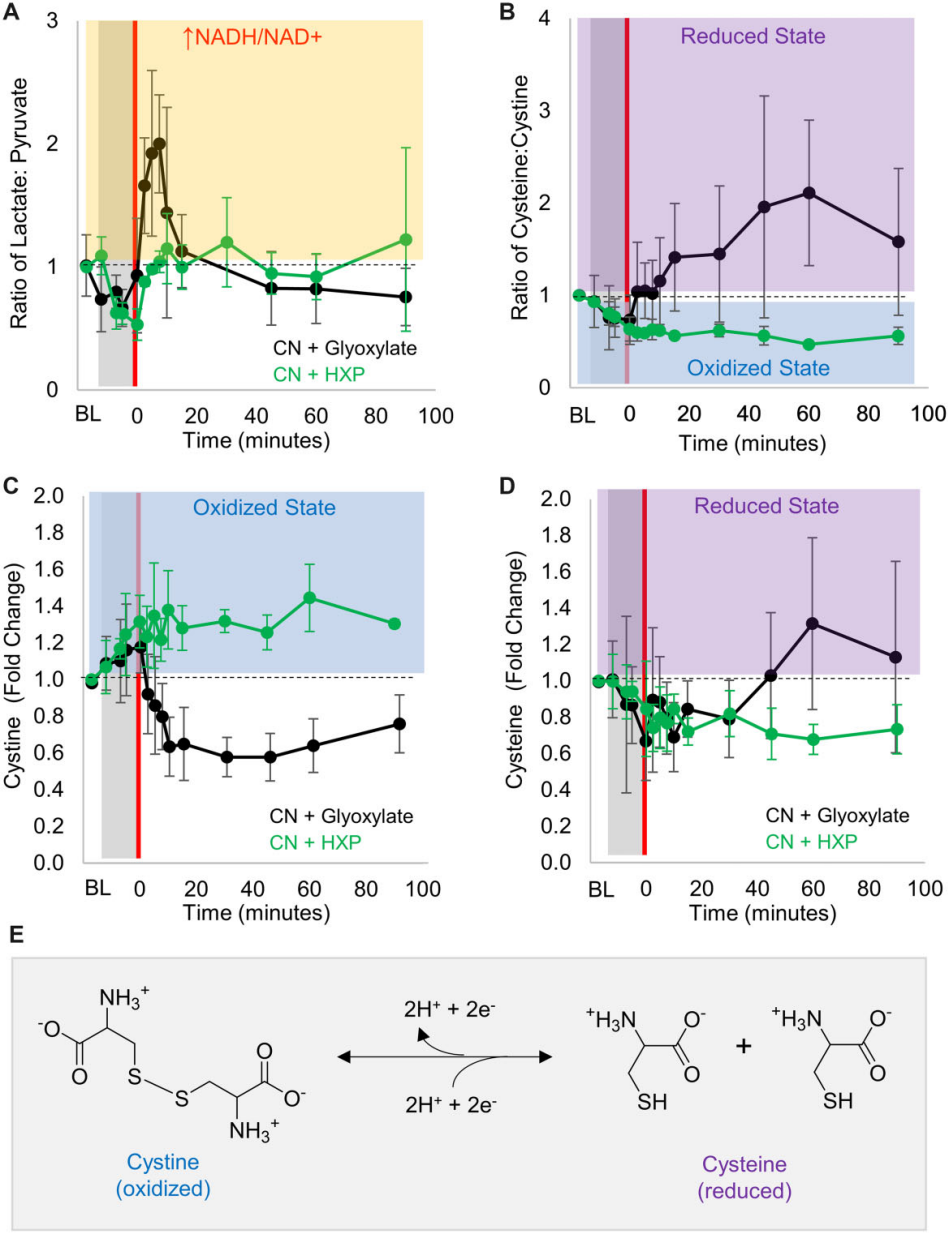
Plasma biomarkers that reflect intracellular and extracellular redox balance improve in glyoxylate-treated animals. In swine treated with cyanide (gray box) and subsequently, at *t* = 0, administered 10 mg/kg glyoxylate IM (black circles; *n* = 6) or 20 mg/kg hexachloroplatinate IM (green circles; *n* = 3), serial plasma samples were taken to measure the (A) ratio of lactate: pyruvate, a circulating marker that is in near equilibrium with cellular NADH: NAD+ ratio, (B) ratio of cysteine: cystine, a plasma biomarker of extracellular oxidative stress, (C) fold change of cystine, and (D) fold change of cysteine. The dashed line represents the baseline of each measurement. Data were normalized to baseline are presented as mean ± standard deviation. E, Diagram of the oxidation/reduction of cysteine/cystine.

To test this hypothesis, we measured the circulating ratio of lactate:pyruvate as it is in near equilibrium with intracellular NADH:NAD+ ratio (Patgiri *et al.*, 2020; Williamson *et al.*, 1967). At the end of the cyanide infusion, plasma lactate:pyruvate decreased by 36% as compared with baseline levels in swine (Figure 5A, gray box; *p* = .01). In conjunction with the elevated lactate levels, this suggests a defect in mitochondrial oxidative phosphorylation during cyanide exposure. Following administration of glyoxylate, lactate:pyruvate rapidly increased from 0.66 ± 0.14 to 1.99 ± 0.39 at 7.5 min post-injection (Figure 5A;*p* = .006), indicating large rapid production of lactate or consumption of pyruvate in response to glyoxylate (which will be described further below). Subsequently, lactate:pyruvate declined to slightly below baseline by 45 min post-injection (0.82 ± 0.29).

To assess the specificity of these findings, we compared the lactate:pyruvate ratios of glyoxylate to that of a known cyanide-chelating agent. Previously, we demonstrated that hexachloroplatinate-DMSO (HCP) binds 3–5 cyanide anions, and intramuscular administration of HCP improves survival and citric acid cycle metabolism in zebrafish, mice, rabbits, and swine (Morningstar *et al.*, 2019; Nath *et al.*, 2017). Here, we demonstrate that HCP (20 mg/kg IM) restores lactate:pyruvate to baseline levels at 7.5 min post-treatment, however, glyoxylate exerted effects on lactate:pyruvate that were distinct from HCP (Figure 5A).

Interestingly, the burst in lactate:pyruvate ratio observed in glyoxylate-treated swine, between *t* = 0 and *t* = 15 min, did not occur in HCP-treated animals (1.99 ± 0.39 vs 1.15 ± 0.28, *p* = .0418; Figure 5A). At the maximum peak in lactate:pyruvate (*t* = 7.5), pyruvate levels declined in glyoxylate-treated animals (*p* = .03) while lactate levels were rising and remained similar between treatment groups (Supplementary Table 3). Following the peak in lactate:pyruvate, that is, from *t* = 7.5 to *t* = 15, lactate production rapidly declined in glyoxylate-treated animals as compared with HCP (*p* = .03) which exhibited continued lactate production (Supplementary Table 3). The effect of glyoxylate on both pyruvate consumption and lactate production, in conjunction with the lactate:pyruvate ratios, suggests that glyoxylate administration in cyanide-poisoned animals alters metabolic pathways in a manner that is distinct from a known cyanide scavenger. These findings also implicate that glyoxylate administration transiently shifts the balance of intracellular NADH/NAD+.

### Glyoxylate improves extracellular redox balance

Extracellular redox state is mainly maintained by the thiol/disulfide pair cysteine (Cys) and cystine (CySS) (Jones *et al.*, 2002; Jones and Go, 2010). The plasma ratio of Cys:CySS is an established biomarker for extracellular redox balance (Go and Jones, 2011; Jones *et al.*, 2002; Moriarty-Craige and Jones, 2004). During cyanide infusion, the extracellular redox environment shifted to an oxidized state as evidenced by decreased plasma Cys:CySS. At the end of the cyanide infusion, Cys:CySS decreased by 27% (*p* = .04) as compared with baseline (Figure 5B). Following IM injection of glyoxylate, the ratio of Cys:CySS increased from 0.73 ± 0.27 to a peak ratio of 2.11 ± 0.78 at 60 min post-injection (Figure 5B;*p* = .01). This indicates that the extracellular redox environment shifted to a reduced state in glyoxylate-treated animals. At the end of the study, Cys:CySS ratio was declining toward baseline while cystine levels remained just below baseline and cysteine levels remained slightly above baseline (Figs. 5B–E).

Next, we sought to compare glyoxylate’s effect on redox state to that of a cyanide-chelating agent. Therefore, we compared Cys:CySS ratios between hexachloroplatinate-treated and glyoxylate-treated pigs. In contrast to glyoxylate, HCP did not improve the cyanide-induced shift to an oxidized state (Figs. 5B–E). Following HCP-administration, Cys:CySS continued to remain below baseline until the end of the study (0.56 ± 0.09 vs 1.00, Figure 5B). This suggests that cyanide chelation alone is not sufficient to ameliorate cyanide-induced oxidative stress in the extracellular compartment in swine.

### Biomarkers of adverse drug reactions do not change during acute glyoxylate exposure

Our metabolite platform measures creatinine and bilirubin, biomarkers of kidney and liver function, respectively. Over a time span of 90 min, it is unlikely to see enzyme increases without cellular necrosis, that is, those detected by comprehensive metabolite panel (ALT, AST, etc.). However, using surrogate metabolite markers of toxicity such as bilirubin or creatinine we can monitor for profound hemolysis or severe renal damage which prompt additional biochemical analyses. To monitor potential acute drug reactions throughout the study, these biomarkers were measured in plasma at baseline and in 13 serial samples. No significant changes in creatinine and bilirubin were observed during this timeframe (Supplementary Figs. 1A and 1B).

### Glyoxylate exhibits a favorable safety profile in rats

We next formally assessed glyoxylate toxicity in rats. Rats (*n* = 6 per group) were treated with a single dose of sodium glyoxylate (60 or 145 mg/kg, IP) or vehicle, and monitored for 5 days. The doses selected for evaluation were based on our previous studies in mice and is the therapeutic dose or 2.5-times the therapeutic dose (Nielson *et al.*, 2022). Allometric scaling from the mouse dose was used to estimate an equivalent dose for rat. On days 1 and 5, blood was collected for comprehensive metabolite panel and complete blood count test (Tables 5–8 and Supplementary Tables 4–7).

**Table 5.** Complete blood count test results in rats 1 day after treatment with 145 mg/kg IP glyoxylate or vehicle

Analyte	Reference range	Vehicle control (*n* = 6)	Glyoxylate (*n* = 6)	***p* value** ^a^
TP	5.3–6.9 g/dl	6.1 ± 0.2	6.5 ± 0.3	.2368
RBC	5.3–7.7 K/µl	7.5 ± 0.3	7.3 ± 0.2	.9589
HCT	36.5–55.5 %	43.0 ± 1.9	41.6 ± 1.3	.9492
HGB	11.0–16.8 g/dl	14.2 ± 0.9	13.8 ± 0.4	.9818
MCV	65.9–73.6 fl	57.4 ± 1.7	57.0 ± 2.6	.9990
MCHC	27.9–30.4 g/dl	33.0 ± 1.8	33.2 ± 0.4	.9986
RDW	N/A %	11.1 ± 0.4	11.6 ± 0.5	.4037
WBC	6.8–14.7 K/µl	10.3 ± 2.6	8.4 ± 0.6	.8153
SEG	3.1–11.4 K/µl	1.7 ± 2.1	1.4 ± 1.3	.9989
LYMPH	81.1–91.5 K/µl	8.3 ± 2.4	6.6 ± 1.1	.9042
EOS	1.1–6.5 K/µl	0.4 ± 0.2	0.2 ± 0.1	.8937
RETIC	N/A K/µl	259 ± 54.6	272.6 ± 58.6	.9992

g/dl, grams per deciliter; M/µl, moles per microliter; fL, femtoliter; K/µl, 1000 per microlitter.

Data presented as mean ± standard deviation.

a *p* values were determined using a Šídák’s multiple comparisons test against the means of vehicle day 1 and glyoxylate treated day 1 rats.

**Table 6.** Complete blood count test results in rats 5 days after treatment with 145 mg/kg IP glyoxylate or vehicle

Analyte	Reference range	Vehicle control (*n* = 6)	Glyoxylate (*n* = 6)	***p* value** ^a^
TP	5.3–6.9 g/dl	6.3 ± 0.2	6.9 ± 0.6	.0298
RBC	5.3–7.7 K/µl	6.9 ± 0.4	7.6 ± 0.7	.0008
HCT	36.5–55.5 %	40.2 ± 3.3	43.9 ± 5.2	.0176
HGB	11.0–16.8 g/dl	13.2 ± 0.9	14.3 ± 1.6	.0162
MCV	65.9–73.6 fl	58.6 ± 1.8	57.6 ± 1.7	.9971
MCHC	27.9–30.4 g/dl	33.0 ± 0.6	32.7 ± 0.3	.9849
RDW	N/A %	12.6 ± 0.7	12.1 ± 0.9	.3134
WBC	6.8–14.7 K/µl	9.6 ± 3	8.3 ± 1.3	.9580
SEG	3.1–11.4 K/µl	1.0 ± 0.9	0.5 ± 0.2	.9702
LYMPH	81.1–91.5 K/µl	8.3 ± 2.7	7.6 ± 1.3	.7742
EOS	1.1–6.5 K/µl	N/A	N/A	N/A
RETIC	N/A K/µl	469.1 ± 59.7	329.6 ± 109.3	.0013

g/dl, grams per deciliter; M/µl, moles per microliter; fl: femtoliter; K/µl, 1000 per microliter.

Data presented as mean ± standard deviation.

a *p* values were determined using a Šídák’s multiple comparisons test against the means of vehicle day 5 and glyoxylate treated day 5 rats.

**Table 7.** Comprehensive metabolic panel results in rats 1 day after treatment with 145 mg/kg IP glyoxylate or vehicle

Analyte	Reference range	Vehicle control (*n* = 6)	Glyoxylate (*n* = 6)	*p* value^*a*^
GLU	50–135 mg/dl	176.3 ± 43.7	172.8 ± 28.0	.9964
BUN	13–28 mg/dl	13.4 ± 2.6	19.7 ± 4.7	.1620
CREA	0.05–0.65 mg/dl	0.5 ± 0.1	0.5 ± 0.1	.9966
PHOS	5.8–11.1 mg/dl	7.5 ± 1.2	6.6 ± 1.1	.9556
CA	5.3–11.6 mg/dl	10.4 ± 0.2	10.4 ± 0.2	.9996
NA	135–146 mmol/l	138.2 ± 0.9	138.3 ± 0.8	.9959
K	4.0–5.9 mmol/l	4.2 ± 0.4	4.2 ± 0.4	>.9999
CL	96–107 mmol/l	100.8 ± 2	101.7 ± 1.5	.6915
CO2	17.7–25.7 mmol/l	24.8 ± 1.5	24.0 ± 1.5	.6145
AGAP	13.3–21.3 mmol/l	16.8 ± 1.4	16.8 ± 1.6	>.9999
TP	5.3–6.9 g/dl	6.1 ± 0.2	6.0 ± 0.3	.9770
ALB	2.9–4.8 g/dl	3.4 ± 0.2	3.4 ± 0.3	.9875
GLOB	1.8–3.0 g/dl	2.7 ± 0.1	2.6 ± 0.1	.6781
A/G	N/A	1.2 ± 0.1	1.3 ± 0.1	.3163
ALT	20–61 IU/l	40.1 ± 7.3	44.7 ± 2.6	.7360
ALKP	16–302 IU/l	202.8 ± 65.8	200.3 ± 37.7	>.9999
GGT	2–3 IU/l	LOD	LOD	N/A
TBIL	N/A mg/dl	LOD	LOD	N/A
CHOL	40–281 mg/dl	94.9 ± 8.7	118 ± 10.8	.0039
AMY	326–2246 IU/l	2045.6 ± 579.6	1916.7 ± 231.7	.9436
LIPA	10–150 IU/l	97.6 ± 22.9	93.2 ± 29.8	.9833

mg/dl, milligrams per deciliter; g/dl, grams per deciliter; mmol/l, millimoles per liter; IU/l, units per liter; LOD, values below the limit of detection for this assay.

Data presented as mean ± standard deviation.

a *p* values were determined using a Šídák’s multiple comparisons test against the means of vehicle day 1 and glyoxylate treated day 1 rats.

**Table 8.** Comprehensive metabolic panel results in rats 5 days after treatment with 145 mg/kg IP glyoxylate

Analyte	Reference range	Vehicle control (*n* = 6)	Glyoxylate (*n* = 6)	*p* value^*a*^
GLU	50–135 mg/dl	174.8 ± 22.5	182.7 ± 47.8	.9614
BUN	13–28 mg/dl	15.6 ± 2.5	24.2 ± 9.6	.0203
CREA	0.05–0.65 mg/dl	0.4 ± 0.1	0.5 ± 0.1	.6685
PHOS	5.8–11.1 mg/dl	6.3 ± 1.3	6.7 ± 0.8	.9867
CA	5.3–11.6 mg/dl	10.4 ± 0.3	10.6 ± 0.3	.6525
NA	135–146 mmol/l	139.3 ± 1.0	142.5 ± 4	.0009
K	4.0–5.9 mmol/l	4.0 ± 0.4	3.9 ± 0.4	.9736
CL	96–107 mmol/l	101.5 ± 1.5	102 ± 2.3	.9307
CO2	17.7–25.7 mmol/l	26.4 ± 1.5	25.5 ± 0.8	.5396
AGAP	13.3–21.3 mmol/l	15.4 ± 1.9	18.9 ± 2.8	.0141
TP	5.3–6.9 g/dl	6.0 ± 0.3	6.4 ± 0.6	.0317
ALB	2.9–4.8 g/dl	3.3 ± 0.2	3.7 ± 0.4	.0043
GLOB	1.8–3.0 g/dl	2.7 ± 0.1	2.7 ± 0.2	.9442
A/G	N/A	1.2 ± 0.1	1.4 ± 0.1	.0948
ALT	20–61 IU/l	43.8 ± 7.1	33 ± 12.4	.1017
ALKP	16–302 IU/l	219.3 ± 55.9	181.2 ± 59.7	.7035
GGT	2–3 IU/l	LOD	LOD	N/A
TBIL	N/A mg/dl	LOD	LOD	N/A
CHOL	40–281 mg/dl	92.3 ± 16	121.5 ± 16	.0003
AMY	326–2246 IU/l	2168.7 ± 565.8	2083.3 ± 379.4	.9959
LIPA	10–150 IU/l	98.5 ± 24	106 ± 38.5	.9261

mg/dl, milligrams per deciliter; g/dl, grams per deciliter; mmol/l, millimoles per liter; IU/l, units per liter; LOD, values below the limit of detection for this assay.

Data presented as mean ± standard deviation.

a *p* values were determined using a Šídák’s multiple comparisons test against the means of vehicle day 5 and glyoxylate treated day 5 rats.

A complete blood count test in rats treated with 60 mg/kg glyoxylate revealed no significant differences in erythrocytes, leukocytes, eosinophils, or lymphocytes on day 1 or 5 post-treatment (Supplementary Tables 4 and 5). At 145 mg/kg glyoxylate, there were no significant differences at day 1 (Table 5). On day 5 post-treatment with 145 mg/kg glyoxylate, total protein was 9.5% greater than vehicle controls (6.9 ± 0.6 vs 6.3 ± 0.2 g/dl, *p* = .0298; Table 6). In addition, concordant RBC traits were elevated ≤10% in glyoxylate-treated animals but were within the reference range for Sprague Dawley rats (RBC: 7.6 ± 0.7 vs 6.9 ± 0.4, *p* = .0008; HCT: 43.9 ± 5.2 vs 40.2 ± 3.3, *p* = .0176; HGB: 14.3 ± 1.6 vs 13.2 ± 0.9, *p* = .0162; Table 6). Markers of red blood cell volume, width, and average cellular hemoglobin concentration (MCV, RDW, and MCHC) were normal. On day 5 as compared with day 1, immature red blood cells (RETIC) were increased in both groups. Together these findings suggest that glyoxylate administration induced mild hemoconcentration which may have been caused by dehydration or osmotic diuresis.

Day 1 post-administration of 60 or 145 mg/kg glyoxylate, there were no significant findings on the comprehensive metabolite panel (Supplementary Table 6 and Table 8). On day 5, at the 60 mg/kg dose, no acute kidney dysfunction was detected in glyoxylate versus vehicle-treated rats as demonstrated by the lack of statistically significant differences in creatinine (0.5 ± 0.1 vs 0.4 ± 0.1 mg/dl, *p* = n.s.), BUN (21.3 ± 4.3 vs 15 ± 3.2 mg/dl, *p* = n.s.), and phosphate levels (5.9 ± 1.5 vs 6.5 ± 1.4 mg/dl, *p* = n.s.; Table 8). Day 5 post-administration of 145 mg/kg dose, albumin was 12% greater than vehicle controls (3.7 ± 0.4 vs 3.3 ± 0.2, *p* = .0043; Table 8), which may be due to hemoconcentration as described earlier. Glyoxylate-treated rats exhibited raised levels of BUN (24.2 ± 9.6 vs 15.6 ± 2.5 mg/dl, *p* = .0203; Table 8). However, additional analytes for kidney dysfunction did not show any statistically significant differences between glyoxylate versus vehicle-treated rats: creatinine (0.5 ± 0.1 vs 0.4 ± 0.1 mg/dl; *p* = n.s.) and phosphate (6.7 ± 0.8 vs 6.3 ± 1.3 mg/dl; *p* = n.s.; Table 8). Further, electrolytes were normal in 145 mg/kg glyoxylate‐treated rats versus vehicle-treated rats at day 5: calcium 10.6 ± 0.3 versus 10.4 ± 0.3 mg/dl (*p* = n.s.), potassium 3.9 ± 0.4 versus 4.0 ± 0.4 mmol/l (*p* = n.s.), and chloride 102 ± 2.3 versus 101.5 ± 1.5 mmol/l (*p* = n.s.). Rats treated with 145 mg/kg glyoxylate exhibited modestly elevated sodium levels at day 5 as compared with vehicle-treated rats (142.5 ± 4 vs 139.3 ± 1.0, *p* = .00009; Table 8). The elevated sodium may be due to the sodium salt formulation of glyoxylate. At the 60 mg/kg glyoxylate dose, rats also exhibited modestly elevated sodium levels (139.5 ± 0.8 vs 137.8 ± 1.0, *p* = .0158); the 2% elevation in sodium was resolved by day 5 (Supplementary Tables 6 and 7). Mildly elevated anion gap (AGAP) that was within the reference range (13.3–21.3 mmol/l) was observed for 145 mg/kg glyoxylate-treated rats at day 5 when compared with the vehicle controls (18.9 ± 2.8 vs 15.4 ± 1.9 mmol/l, *p* = .0141; Table 8); animals exhibited normal chloride (102.0 ± 2.1 vs 100.5 ± 2.1 mmol/l, *p* = n.s.) and bicarbonate values (25.3 ± 1.9 vs 25.3 ± 0.8 mmol/l, *p* = n.s.) (Car *et al.*, 2005), suggesting a possible metabolic acidosis likely due to the acids derived from glyoxylate. Liver (ALT and ALKP) and pancreatic enzymes (AMY and LIPA) were not significantly altered by either dose of glyoxylate and were within the normal reference range of Sprague Dawley rats (Tables 7 and 8 and Supplementary Tables 6 and 7). Cumulatively, these clinical findings demonstrate that 60–145 mg/kg glyoxylate is well tolerated by rats.

## Discussion

Cyanide blockade of the mitochondrial electron transport chain prevents ATP generation from oxidative phosphorylation. Cellular respiration is forced to shift from aerobic to anaerobic, resulting in elevated lactate concentration that is driven by LDH reduction of pyruvate to lactate coupled to oxidation of NADH to NAD+. During cyanide exposure, NADH is consumed via anaerobic glycolysis in the cytoplasm, whereas in the mitochondria, complex I is unable to accept electrons from NADH and hence regenerate NAD+. Glyoxylate is an endogenous intermediary metabolite that generates both NADH and NAD+ cofactors via its capability to be either oxidized or reduced (Baker *et al.*, 2004; Duncan and Tipton, 1969; Mdluli *et al.*, 2005; Romano and Cerra, 1969; Warren, 1970). In addition, NADH and NAD+ balances in the cytoplasm and mitochondria are modulated via the metabolic interplay between the subcellular metabolism of glyoxylate (Supplementary Figure 2). Further, glyoxylate has ample capacity to reversibly bind cyanide in the form of a cyanohydrin (Nielson *et al.*, 2022). Here we demonstrate in our large animal swine model of lethal cyanide poisoning that administration of glyoxylate improves survival, clinical outcomes, citric acid cycle metabolism, and redox balance.

Glyoxylate is largely metabolized by the liver and its anabolites are glycine, oxalate, and glycolate (Holmes and Assimos, 1998; Takayama *et al.*, 2003; Wanders and Waterham, 2006). Importantly, the specific products derived from glyoxylate metabolism are tightly correlated with the subcellular localization of glyoxylate and the enzymatic activity of the corresponding dehydrogenases and oxidases in that subcellular compartment (Supplementary Figure 2) (Noguchi *et al.*, 1978). The liver-specific peroxisomal enzyme alanine-glyoxylate aminotransferase converts glyoxylate to glycine (Wanders and Waterham, 2006); whereas the peroxisomal/cytosolic enzyme glycolate oxidase converts glyoxylate to oxalate. The cytosolic/mitochondrial enzyme glyoxylate reductase (GRHPR) and the cytoplasmic (predominantly)/mitochondrial enzyme LDH can both oxidize glyoxylate to oxalate or reduce it to glycolate (Baker *et al.*, 2004; Duncan and Tipton, 1969; Mdluli *et al.*, 2005; Romano and Cerra, 1969; Warren, 1970). Cumulatively, these enzymes regulate the subcellular metabolism of glyoxylate. Previously, we demonstrated that glycolate and oxalate do not provide any protection against cyanide poisoning (Nielson *et al.*, 2022). Therefore, glycolate and oxalate per se are not the mechanism of protection. Notably, glyoxylate metabolism is coupled to oxidation-reduction of NADH/NAD+ or NADPH/NADP+, in addition to the generation of pyruvate (Supplementary Figure 2). Therefore, the rapid absorption of glyoxylate and its cellular metabolism may lead to regeneration of essential co-factors that were depleted during cyanide intoxication.

Intracellular NADH:NAD+ ratio is in near equilibrium with the circulating ratio of lactate:pyruvate (Patgiri *et al.*, 2020; Williamson *et al.*, 1967). Following administration of glyoxylate, we observed a burst in lactate:pyruvate ratio (Figure 5A;*t* = 0 to *t* = 15), which was not observed in animals treated with HCP, a cyanide chelator. At the peak ratio of lactate:pyruvate (*t* = 7.5) in glyoxylate-treated animals, pyruvate levels declined while lactate levels were rising. The rise in lactate was also observed in HCP-treated animals, however the reduction in pyruvate was not observed (Supplementary Table 3). Subsequent to the maximum peak in lactate:pyruvate, that is, from *t *= 7.5 to *t* = 15, lactate production rapidly declined in glyoxylate-treated animals as compared with HCP-treated animals which exhibited continued lactate production. The effect of glyoxylate on both pyruvate consumption and lactate production, in conjunction with the lactate:pyruvate ratios, suggests that glyoxylate administration in cyanide-poisoned animals transiently shifts the balance of intracellular NADH/NAD+. Moreover, this pattern was not observed in HCP-treated animals indicating that glyoxylate alters metabolic pathways in a manner that is distinct from a known cyanide scavenger. During this same timeframe (*t* = 5 to *t* = 15), second-span TCA cycle metabolites were consumed and, subsequently, continued to decline toward baseline in glyoxylate-treated animals (Figure 3). This suggests a shift from glycolysis to activation of citric acid cycle metabolism. The burst in citric acid cycle activity during the apneic phase of cyanide toxicity may contribute to the beneficial effects of glyoxylate on clinical outcomes and survival. Concordantly, our previous studies in rabbits demonstrated that glyoxylate rapidly reversed cyanide-induced reduction in cytochrome C oxidase redox state (Nielson *et al.*, 2022), which is consistent with our data in swine showing rapid reactivation of the TCA cycle. In summary, our data indicates that glyoxylate’s mechanism of protection in swine is, in part, due to the generation of essential metabolic cofactors and reactivation of TCA cycle metabolism.

In perspective, the 2 FDA-approved cyanide antidotes act by stoichiometric chelation or detoxification of cyanide (Borron and Baud, 2012). Upon administration, these antidotes bind cyanide anions and thereby halt cyanide-induced toxicity but they do not reverse the biological damage that has already occurred. Interestingly, compounds that specifically ameliorate cyanide-induced redox imbalances, independent of chelation, have been shown to be an effective strategy to counteract cyanide toxicity in animal models (Haouzi *et al.*, 2019). This may be because redox homeostasis is tied to cellular metabolism. Our findings indicate that glyoxylate impacts redox balances. In addition to changes in intracellular redox balances (lactate:pyruvate), glyoxylate modulates extracellular redox balances. The cysteine/cystine redox couple are a key mechanism that regulates extracellular redox balance (Go and Jones, 2011; Jones *et al.*, 2002; Jones and Go, 2010; Moriarty-Craige and Jones, 2004). Glyoxylate reversed cyanide-induced oxidative stress in the extracellular compartment as evidenced by reversing the plasma ratio of Cys:CySS (Figure 5C). By contrast, a cyanide chelating agent (HCP) was not sufficient to ameliorate cyanide-induced oxidative stress in the extracellular compartment (Figure 5D). These findings suggest that glyoxylate’s mechanism of action may also include modulation of extracellular redox state.

Our pharmacokinetic analyses in swine administered glyoxylate, demonstrated a sharp rise in circulating glyoxylate followed by its rapid clearance, concomitant with increased and sustained levels of circulating oxalate and no significant changes in glycine (albeit plasma glycine levels are in the hundreds of micromolar range making it challenging to identify small changes in its concentration) (Figure 4). Oxalate is an end-product metabolite that is excreted by the kidney. The conversion of glyoxylate to oxalate may lead to hyperoxaluria. In glyoxylate-treated swine, the *C*_max_ of oxalate was 8.0 ± 0.5 µM at 65 min post-treatment. Patients with type 2 diabetes have been shown to exhibit plasma oxalate concentrations as high as 25 µM (Nikiforova *et al.*, 2014). In patients with Primary Hyperoxaluria Type 1—a genetic mutation in alanine-glyoxylate aminotransferase—plasma oxalate concentrations range from 3.7 to 14.6 μM (Perinpam *et al.*, 2017). In both healthy and diseased patients, oxalate is excreted by the kidneys. In patients with Primary Hyperoxaluria Type 1, increased fluids and oral potassium citrate are used to help prevent calcium oxalate crystals from forming. Primary Hyperoxaluria Type 1 is a life-long disease with chronic bouts of increased plasma and urinary oxalate levels, whereas in cyanide-poisoned individuals, the oxalate derived from single dose administration of glyoxylate is anticipated to be excreted as it is within the concentration of that observed in patients with chronic diseases that experience hyperoxaluria.

In a mass casualty scenario, the preferred method of countermeasure delivery is intramuscular using an autoinjector (Jett and Yeung, 2010). In this study, swine were administered glyoxylate at 10 mg/kg in a total volume of 3.5 ml of phosphate buffer. Therefore, using an allometric scaling factor of 1.1 (U.S. Department of Health and Human Services, 2005) and the current formulation (140 mg/ml 0.1 M phosphate buffer, pH 7.5), the glyoxylate dose and buffer volume required for humans would be 11 mg/kg in 3.8 ml. Several autoinjectors in development for cyanide medical countermeasure delivery have a volume limit of 2.5 ml (Mesa, 2022). However, the devices that will be used to administer medical countermeasures for toxicants are routinely developed in collaboration with industry partners around the drug that is to be administered. Therefore, having determined the efficacious dose in swine—and the scaled human dose—future studies will include improving the pharmaceutics (eg, reducing the osmotic strength of formulation) and physiochemical stability studies of a human dose equivalent formulation which will be designed around an autoinjector with volume limitations.

This study has some limitations. First, the animals were anesthetized per IACUC requirement to reduce pain and suffering. Isoflurane may have confounding effects; but we adjusted the isoflurane to be the minimum amount needed for sedation. In addition, vehicle controls were also sedated. Moreover, our model is well-characterized and widely used to determine countermeasure efficacy (Bebarta *et al.*, 2014; *al.*, 2017; Chan *et al.*, 2022; Hendry-Hofer *et al.*, 2020; *al.*, 2022; Morningstar *et al.*, 2019). Second, our metabolite platform does not measure glycolate, therefore we do not know the amount of glycolate produced following glyoxylate administration. Third, we measured metabolites in plasma, therefore we do not know the tissue source of changes in the levels of citric acid cycle metabolites. Also, we measured circulating lactate:pyruvate which is in near equilibrium with intracellular NADH/NAD+ (Patgiri *et al.*, 2020; Williamson *et al.*, 1967), however a panel of validated markers that inform on tissue-specific NADH/NAD+ ratios does not exist. Future studies are required to determine the tissue-specific site of action. Fourth, an alternative potential mechanism by which glyoxylate improves rebreathing and blood oxygen levels is by an effect on the carotid body and ventilatory responses. It has been shown that ROS, NADH, pH, and other factors affect voltage-dependent potassium channels on the carotid body thereby modulating chemostimulation and ventilatory responses (Holmes *et al.*, 2022). Future studies are required to determine if glyoxylate-mediated modulation of NAD+/NADH and/or redox state, or other actions of glyoxylate in the carotid body influence carotid body function. Fifth, interestingly, endogenous levels of glyoxylate decreased during cyanide infusion. We do not know the metabolic fate of endogenous glyoxylate consumption during cyanide infusion. Future studies with isotope-labeled glyoxylate will be required to determine the metabolic fate of glyoxylate during cyanide poisoning. Sixth, this study did not assess long-term outcomes in surviving animals. Future studies will evaluate neurobehavioral function in cyanide-treated swine administered glyoxylate as a countermeasure.

## Impact statement

Intramuscular administration of the endogenous metabolite glyoxylate in cyanide-poisoned swine improves survival, clinical physiological parameters, citric acid cycle metabolism, and redox balances. By both binding cyanide and addressing the biochemical sequela of cyanide exposure, glyoxylate may expand the current antidote toolkit beyond chelation of cyanide to ameliorating the cellular effects of cyanide. Moreover, as the toxidrome of several metabolic poisons involves redox balance, glyoxylate may be an efficacious medical countermeasure for other metabolic poisons, including hydrogen sulfide and sodium azide.

## Supplementary data

Supplementary data are available at *Toxicological Sciences* online.

## Author contributions

Study design—A.K.N. and V.S.B; Experimental investigation—C.C.S., S.Z., T.B.H., and X.S.; Data analysis—A.K.N., C.C.S., G.B., G.K., J.R., M.B., S.M., T.B.H., V.S.B., and X.S.; Writing—original draft: A.K.N.; Writing—review and editing: A.K.N., C.A.M., G.B., G.K., J.D., J.R., M.B., R.E.G., R.T.P., S.M., T.B.H., and V.S.B.; Project administration—A.K.N and V.S.B.; Funding and resources—A.K.N., J.D., R.E.G., and V.S.B.

## Funding

The National Institutes of Health CounterACT program (U54NS112107); National Institute of Allergy and Infectious Diseases.

## Declaration of conflicting interests

The authors declared no potential conflicts of interest with respect to the research, authorship, and/or publication of this article.

## Supplementary Material

kfac116_Supplementary_Data
