# Supplementary material for: Intramuscular administration of glyoxylate rescues swine from lethal cyanide poisoning and ameliorates the biochemical sequalae of cyanide intoxication
Source: Toxicol Sci. 2022 Nov 3;191(1):90–105. doi: 10.1093/toxsci/kfac116 (PMC9887668; doi:10.1093/toxsci/kfac116)
Supplement: kfac116_Supplementary_Data [file kfac116_supplementary_data.docx]

**Supplementary Material**

Intramuscular Administration of Glyoxylate Rescues Swine from Lethal Cyanide Poisoning and Ameliorates the Biochemical Sequalae of Cyanide Intoxication

Vik S. Bebarta^1^, Xu Shi^2^, Shunning Zheng^2^, Tara B. Hendry-Hofer^1^, Carter C. Severance^1^, Matthew M. Behymer^3^, Gerry R. Boss^4^, Sari Mahon^5^, Matthew Brenner^5^, Gregory T. Knipp^3^, Vincent Jo Davisson^6^, Randall T. Peterson^7^, Calum A. MacRae^8^, Jared Rutter^9^, Robert E. Gerszten^2,10,11^, and Anjali K. Nath^2,10,11,12^

^1^Department of Emergency Medicine, University of Colorado School of Medicine, Aurora, CO 80045, USA.

^2^Department of Cardiology, Beth Israel Deaconess Medical Center, Boston, MA 02115, USA.

^3^Department of Industrial and Physical Pharmacy, Purdue University, West Lafayette, Indiana 47907, USA.

^4^Department of Medicine, University of California, San Diego, CA 92093, USA.

^5^Beckman Laser Institute and Department of Medicine, University of California, Irvine, CA 92697, USA.

^6^Department of Industrial and Physical Pharmacy, Purdue University, West Lafayette, Indiana 47907, USA.

^7^Department of Pharmacology and Toxicology, College of Pharmacy, University of Utah, Salt Lake City, UT 84112 USA.

^8^Division of Cardiovascular Medicine, Brigham and Women's Hospital, Boston, MA 02115, USA.

^9^Department of Biochemistry and Howard Hughes Medical Institute, University of Utah, Salt Lake City, USA.

^10^Broad Institute, Cambridge, MA 02142, USA.

^11^Harvard Medical School, Boston, MA 02115, USA.

^12^To whom correspondence should be addressed: Anjali K. Nath, anath1@bidmc.harvard.edu

**SUPPLEMENTARY METHODS**

**Mass spectrometry.** EDTA blood samples were collected and immediately centrifuged to separate cellular material from plasma. Aliquots of plasma were stored at –80°C until analysis. Plasma samples (30 µL) were deproteinized using 70 µL of acetonitrile/methanol (75:25; v/v) containing stable isotope-labeled and deuterated internal standards 25 μM thymine-d_4_, 10 μM inosine-^15^N_4_, 10 μM citrulline-d_7_, 25 μM phenylalanine-d_8_, and 10 μM valine-d_8_. Samples were vortexed briefly and then centrifuged (10,000 g for 10 minutes at 4ºC). Supernatants were transferred to glass autosampler vials containing glass inserts (MicroSolv) and subjected to LC-MS/MS analysis. The samples were separated using a 2.1 × 100 mm 3.5-μm Xbridge amide column (Waters). Mobile phase A was 95:5 (v/v) water/acetonitrile, with 20 mM ammonium acetate and 20 mM ammonium hydroxide (pH 9.5). Mobile phase B was acetonitrile. For amide-negative mode, the chromatography system consisted of a 1260 Infinity autosampler (Agilent) connected to a 1290 Infinity HPLC binary pump system (Agilent). The initial conditions were 0.25 ml/min of 85% mobile phase B, followed by a linear gradient to 35% mobile phase B over 6 minutes. This was followed by a linear gradient to 2% mobile phase B over 0.5 minutes held for an additional 0.5 minutes and then a 0.5-minute gradient return to 85% mobile phase B. Column equilibration was continued for 4.5 minutes at 0.5 ml/min for a total cycle time of approximately 12.5 minutes. The column compartment was maintained at 30°C. Using purified reference standards, metabolites were optimized for negative-mode detection on a 6490 QQQ (Agilent) triple-quadrupole mass spectrometer equipped with an electrospray ionization source. Multiple reaction monitoring (MRM) transitions for each compound (**Supplementary Table 1**) were assessed for sensitivity, selectivity, and retention time in the pooled plasma matrix with and without the spiked reference standard. The MS settings were as follows: sheath gas temperature, 400°C; sheath gas flow, 12 l/min; drying gas temperature, 290°C; drying gas flow, 15 l/min; capillary, 4,000 V; nozzle pressure, 30 psi; nozzle voltage, 500 V; and delta EMV, 200 V. LC-MS data were quantified using MassHunter Quantitative Analysis Software (Agilent). All metabolite peaks were manually reviewed for peak quality in a blinded manner and compared against a known standard to confirm identity. Pooled plasma was interspersed throughout the run at regular intervals to monitor mass spectrometry performance (temporal drift) and the variability of each analyte.

For positive mode, plasma samples (10 μL) were deproteinized using 90 μL of methanol/acetonitrile (75:25; v/v) containing isotopically labeled internal standards (10 μM valine-d_8_ and 10 μM phenylalanine-d_8_). Samples were vortexed briefly and then centrifuged (10,000 g for 10 minutes at 4ºC). Supernatants were transferred to glass autosampler vials containing glass inserts (MicroSolv) and subjected to LC-MS/MS analysis. The samples were separated using a 2.1 × 150 mm 3.5 μm Atlantis HILIC column (Waters). The chromatography system included an Agilent 1200 series LC with a CTC PAL Autosampler. Mobile phase A was of 10 mM ammonium formate with 0.1% formic acid (v/v). Mobile phase B was acetonitrile with 0.1% formic acid (v/v). The column was eluted isocratically with 5% mobile phase A and 95% mobile phase B for one minute followed by a linear gradient to 60% mobile phase A and 40% mobile phase B over 10 minutes. MS analyses were carried out using electrospray ionization and MRM scans in the positive ion mode on a 4000 QTRAP triple quadrupole mass spectrometer (Applied Biosystems/Sciex). MRM transitions were optimized for each species (**Supplementary Table 1**) using authentic reference standards. The MS settings were as follows: source temperature, 450°C; ion spray voltage, 5,000 V; CAD gas, 10; CUR gas, 20. LC‐MS data were quantified using Multiquant software (Applied Biosystems/Sciex). All metabolite peaks were manually reviewed for peak quality in a blinded manner and compared against a known standard to confirm identity. Pooled plasma was interspersed throughout the run at regular intervals to monitor mass spectrometry performance (temporal drift) and the variability of each analyte.

To monitor analytical reproducibility throughout the run, pooled plasma reference samples were interspersed throughout the run. Deuterated internal standards exhibited a coefficient of variation (CV) of 3.6% to 5.6% (**Supplementary Table 1**). Measurement variation is specific to each analyte; the analytes measured in our study exhibited CVs ranging from 2.0% to 12.1% (**Supplementary Table 1**). These are well below the cutoff value of 20-30% that is used in the field of mass spectrometry-based metabolomics (Dunn et al. 2011).

**Hexachloroplatinate (HCP) administration.** To 1000 mg sodium HCP (Sigma Aldrich, cat# 288152), we added 1 mL of DMSO and vortexed the tube for 2 minutes. Next 10.5 mL of Ca^2+^, Mg^2+^‐free PBS solution (at 95°C) was added to the tube which was further vortexed for 2 minutes. This solution was delivered intramuscularly into the gluteal muscle in swine.


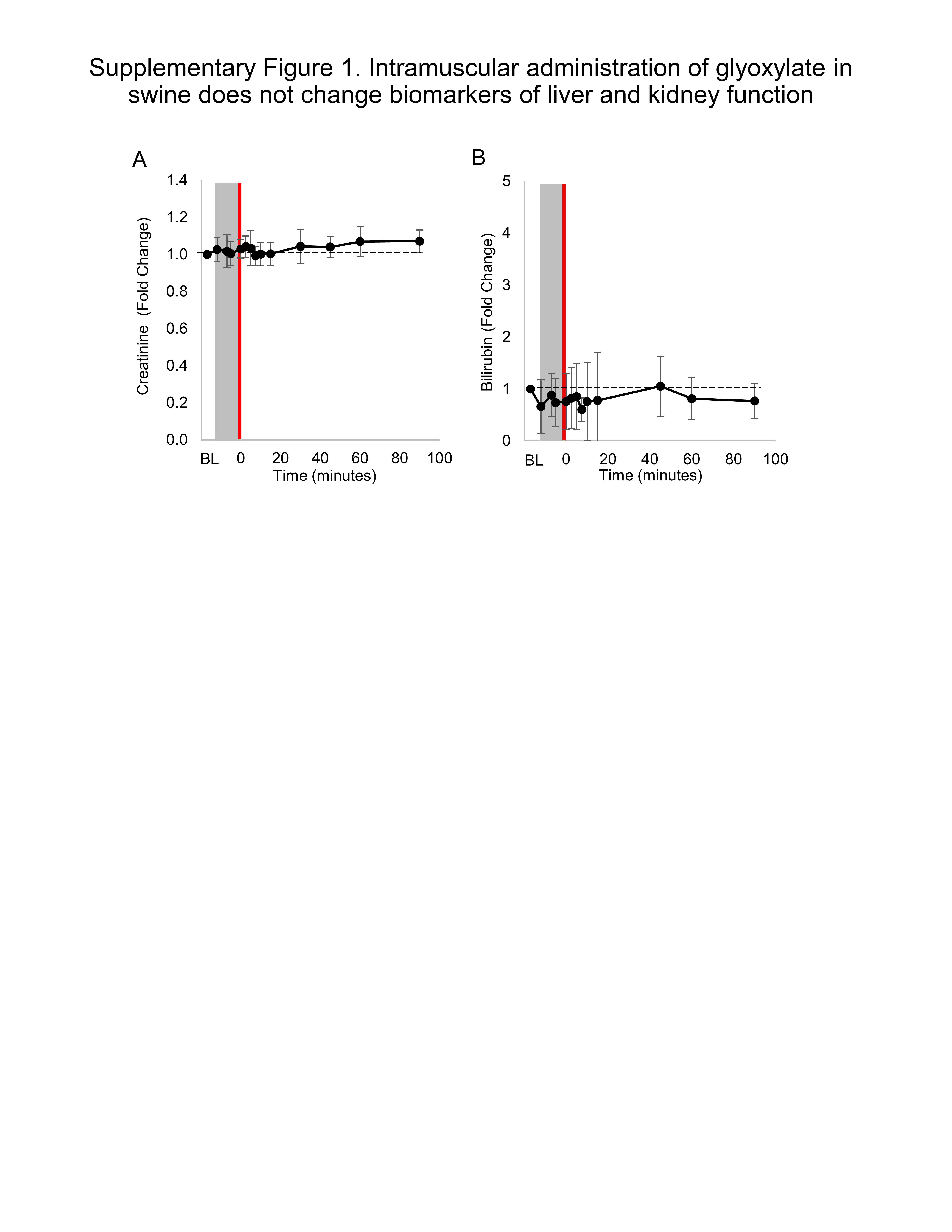


**Supplementary Figure 1. Intramuscular administration of glyoxylate in swine does not change biomarkers of liver and kidney function.** Plasma levels of **A)** creatinine (kidney biomarker) and **B)** bilirubin (liver biomarker) in swine treated with cyanide (gray box) and subsequently administered 10 mg/kg glyoxylate IM at t=0. Data were normalized to baseline are presented as mean ± standard deviation. The dashed line represents the baseline of each measurement.


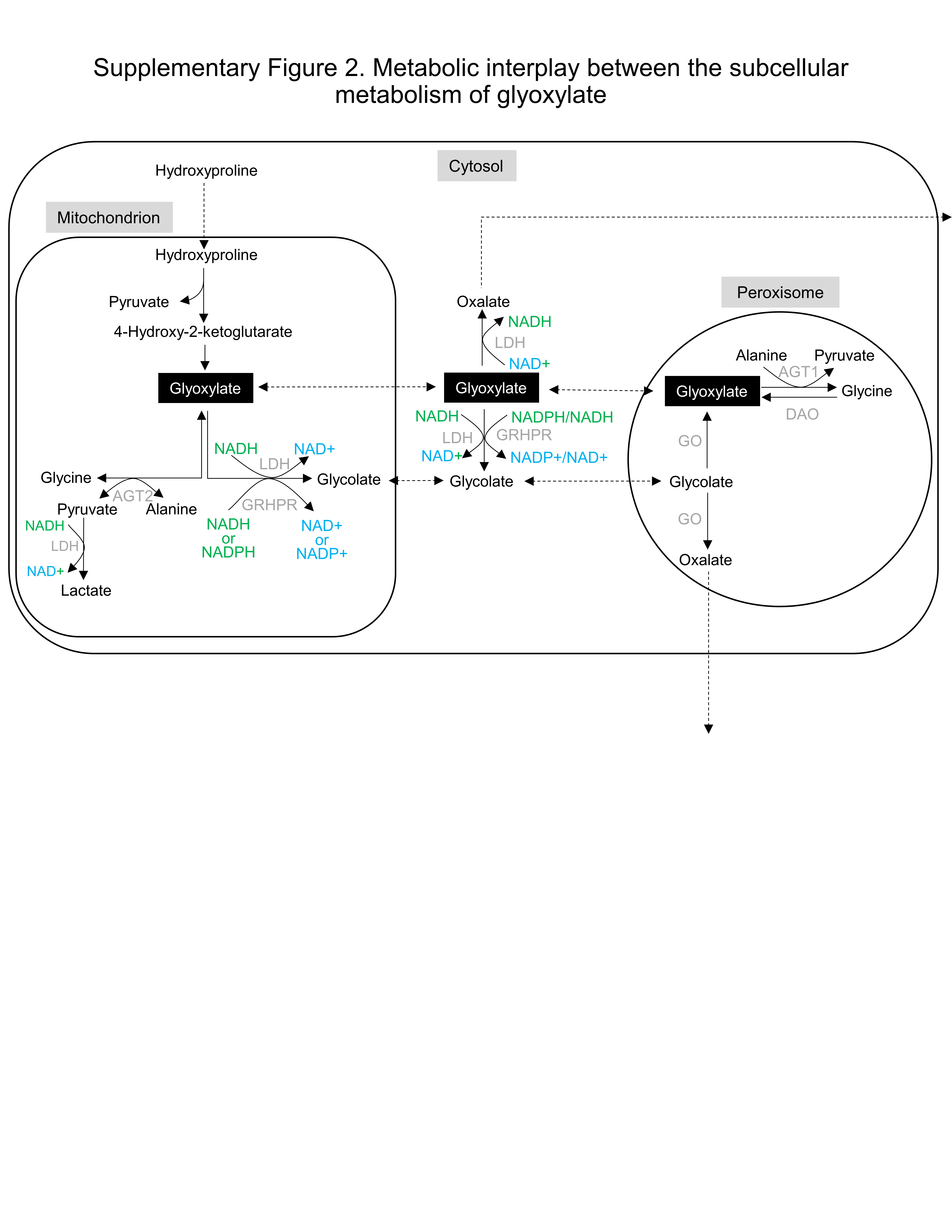


**Supplementary Figure 2. Metabolic interplay between the subcellular metabolism of glyoxylate.** The specific pathways of glyoxylate metabolism are segregated by the activity, concentration and localization of the required metabolic enzymes and their cofactors to different subcellular compartments. Many of these reactions are coupled with the reduction or oxidation of cofactors. Notably, lactate dehydrogenase is capable of both oxidation and reduction of glyoxylate, and GRHPR competes with LDH for the cofactor NADH. LDH lactate dehydrogenase, GRHPR glyoxylate reductase/hydroxypyruvate reductase, GO glycolate oxidase, AGT alanine-glyoxylate transaminase, DAO d-amino acid oxidase, NAD+/NADH nicotinamide adenine dinucleotide, NADP+/NADPH nicotinamide adenine dinucleotide phosphate, and FAD/FADH_2_ flavin adenine dinucleotide.

**Supplementary Table 1. Multiple reaction monitoring transitions of compounds and their coefficient of variation in pooled plasma.**

|  | Ionization Mode | | Q1  Precursor (m/z) | Q3  Product (m/z) | | Collision Energy (eV) | | Metabolite  Name | Coefficient of  Variation |
| --- | --- | --- | --- | --- | --- | --- | --- | --- | --- |
|  | Negative | 181.22 | | 138.2 | 6 | | Citrulline-d_7_ | | 4.5 |
|  | Negative | 271.2 | | 138.9 | 22 | | Inosine-^15^N_4_ | | 5.6 |
|  | Positive | 174.2 | | 128 | 19 | | Phenylalanine-d_8_ | | 3.6 |
|  | Negative | 161.1 | | 113.1 | 2 | | Glucose | | 6.6 |
|  | Negative | 87 | | 43 | 14 | | Pyruvic acid | | 3.8 |
|  | Negative | 89 | | 43.2 | 16 | | Lactic acid | | 2.0 |
|  | Negative | 131 | | 87 | 14 | | Oxaloacetate | | 4.7 |
|  | Negative | 191 | | 111 | 15 | | Citric acid-Isocitratic acid | | 4.5 |
|  | Negative | 173.05 | | 85 | 17 | | Aconitate | | 5.2 |
|  | Negative | 145 | | 101 | 13 | | α-Ketoglutarate | | 4.1 |
|  | Negative | 117 | | 73 | 12 | | Succinate | | 6.4 |
|  | Negative | 115 | | 71 | 13 | | Fumarate | | 6.9 |
|  | Negative | 133 | | 115 | 14 | | Malate | | 4.1 |
|  | Negative | 73 | | 45 | 22 | | Glyoxylic acid | | 4.3 |
|  | Negative | 89 | | 61 | 18 | | Oxalic acid | | 8.7 |
|  | Positive | 76.1 | | 30.5 | 18 | | Glycine | | 8.0 |
|  | Positive | 132.004 | | 68.2 | 19 | | Hydroxyproline | | 5.6 |
|  | Positive | 241.002 | | 74 | 32 | | Cystine | | 8.2 |
|  | Positive | 122.1 | | 59.1 | 29 | | Cysteine | | 8.0 |
|  | Positive | 114 | | 44.2 | 19 | | Creatinine | | 4.6 |
|  | Negative | 583.2 | | 285 | 36 | | Bilirubin | | 12.1 |

m/z: mass/charge; eV: electron volt.

**Supplementary Table 2. Pharmacokinetic parameters of glyoxylate in swine.**

| **Parameter** | **Unit** | **Glyoxylate (n=5)** | **Oxalate (n=5)** |
| --- | --- | --- | --- |
| AUC | µmol*min/L | 826 ± 208 | 536 ± 111 |
| C_max_ | µmol/L | 16.2 ± 6.1 | 8.0 ± 0.5 |
| T_max_ | minutes | 9.5 ± 5.4 | 65.0 ± 22.6 |
| t_1/2_ | minutes | 60.7 ± 28.2 | N/A |

AUC: area under the curve; C_max_: maximum plasma concentration of metabolite; T_max_: time to reach maximum plasma concentration of metabolite; t_1/2_: time for plasma concentration of metabolite to reduce to half its maximum concentration; µmol*min/L: micromoles x minutes/litter; µmol/L: micromoles/litter.

**Supplementary Table 3. Plasma pyruvate and lactate levels at the timepoint of peak lactate-to-pyruvate value and 15 minutes post-treatment in cyanide-poisoned animals treated with vehicle, glyoxylate, or a cyanide-chelating agent.**

| **Timepoint** | **Metabolite** | **Glyoxylate** | **HXP** | **P-Value** |
| --- | --- | --- | --- | --- |
| End of CN Infusion | Pyruvate | 2.4 ± 1.0 | 2.3 ± 0.3 | n.s. |
| End of CN Infusion | Lactate | 1.6 ± 1.7 | 1.7 ± 0.8 | n.s. |
| 7.5 min (i.e. Peak Lac:Pyr) | Pyruvate | 1.7 ± 0.3 | 3.3 ± 1.1 | 0.03 |
| 7.5 min (i.e. Peak Lac:Pyr) | Lactate | 3.5 ± 0.9 | 3.7 ± 0.7 | n.s. |
| 15 min Post-Tx | Pyruvate | 2.1 ± 0.6 | 3.5 ± 0.5 | n.s. |
| 15 min Post-Tx | Lactate | 2.0 ± 0.5 | 3.6 ± 0.4 | 0.03 |

HXP: hexacholoroplatinate (n=3); Gly: glyoxylate (n=6); Lac:Pyr: lactate to pyruvate ratio; Post-Tx: post-treatment; n.s.: not statistically significant.

**Supplementary** **Table 4. Complete blood count test results in rats 1 day after treatment with 60 mg/kg IP glyoxylate or vehicle.**

| **Analyte** | **Units** | | **Vehicle Control (n=6)** | **Glyoxylate (n=6)** | ***P* value^†^** |
| --- | --- | --- | --- | --- | --- |
| TP (R) | | 5.3-6.9 g/dL | 6.1 ± 0.3 | 6.5 ± 0.5 | 0.1347 |
| RBC | | 5.3-7.7 K/µL | 7.4 ± 0.1 | 7.6 ± 0.4 | 0.4509 |
| HCT | | 36.5-55.5 % | 42.2 ± 1.6 | 43.9 ± 2 | 0.4128 |
| HGB | | 11.0-16.8 g/dl | 13.6 ± 1.0 | 14.5 ± 0.6 | 0.1977 |
| MCV | | 65.9- 73.6 fL | 57.2 ± 1.5 | 57.6 ± 2.1 | 0.9440 |
| MCHC | | 27.9 - 30.4 g/dL | 32.2 ± 2.5 | 33.1 ± 0.7 | 0.4726 |
| RDW | | N/A % | 11.1 ± 0.4 | 11.0 ± 0.1 | 0.9806 |
| WBC | | 6.8-14.7 K/µL | 11.2 ± 2.9 | 9.5 ± 3.6 | 0.6528 |
| SEG | | 3.1-11.4 K/µL | 1.3 ± 0.8 | 1.0 ± 0.4 | 0.4749 |
| LYMPH | | 81.1-91.5 K/µL | 9.6 ± 2.4 | 8.3 ± 3.8 | 0.7886 |
| EOS | | 1.1-6.5 K/µL | 0.4 ± 0.2 | 0.2 ± 0.2 | 0.5189 |
| RETIC | | N/A K/µL | 266.9 ± 60.1 | 234.8 ± 42.3 | 0.5381 |

g/dL: grams per deciliter; M/µL: moles per microliter; fL: femtoliter; K/µL: 1,000 per microlitter.

Data presented as mean ± standard deviation.

**^†^**P values were determined using a Šídák's multiple comparisons test against the means of vehicle day 1 and glyoxylate treated day 1 rats.

**Supplementary Table 5. Complete blood count test results in rats 5 days after treatment with 60 mg/kg IP glyoxylate or vehicle.**

| **Analyte** | **Units** | | **Vehicle Control (n=6)** | **Glyoxylate (n=6)** | ***P* value^†^** |
| --- | --- | --- | --- | --- | --- |
| TP (R) | | 5.3-6.9 g/dL | 6.4 ± 0.2 | 6.5 ± 0.1 | 0.9353 |
| RBC | | 5.3-7.7 K/µL | 6.8 ± 0.3 | 6.8 ± 0.5 | 0.9970 |
| HCT | | 36.5-55.5 % | 39.9 ± 2.9 | 40.9 ± 3.5 | 0.8276 |
| HGB | | 11.0-16.8 g/dl | 13.1 ± 0.8 | 13.4 ± 1 | 0.8069 |
| MCV | | 65.9- 73.6 fL | 58.8 ± 2.2 | 60.4 ± 2.1 | 0.3886 |
| MCHC | | 27.9 - 30.4 g/dL | 32.8 ± 0.5 | 32.8 ± 0.6 | 0.9828 |
| RDW | | N/A % | 12.5 ± 0.5 | 12.9 ± 0.6 | 0.4071 |
| WBC | | 6.8-14.7 K/µL | 8.9 ± 2.9 | 9.7 ± 2.9 | 0.9147 |
| SEG | | 3.1-11.4 K/µL | 0.6 ± 0.3 | 1.0 ± 0.5 | 0.4163 |
| LYMPH | | 81.1-91.5 K/µL | 8.3 ± 3.1 | 8.6 ± 2.4 | 0.9882 |
| EOS | | 1.1-6.5 K/µL | 0.2 ± 0.3 | 0.3 ± 0.3 | 0.9946 |
| RETIC | | N/A K/µL | 453.0 ± 57.3 | 512.4 ± 21.5 | 0.1479 |

g/dL: grams per deciliter; M/µL: moles per microliter; fL: femtoliter; K/µL: 1,000 per microlitter.

Data presented as mean ± standard deviation.

**^†^**P values were determined using a Šídák's multiple comparisons test against the means of vehicle day 5 and glyoxylate treated day 5 rats.

**Supplementary Table 6. Comprehensive metabolic panel results in rats 1 day after treatment with 60 mg/kg IP glyoxylate or vehicle.**

| **Analyte** | **Reference Range** | | **Vehicle Control (n=6)** | **Glyoxylate (n=6)** | ***P* value^†^** |
| --- | --- | --- | --- | --- | --- |
| GLU | | 50-135 mg/dL | 205.2 ± 45.6 | 172.2 ± 39.9 | 0.2299 |
| BUN | | 13-28 mg/dL | 15.2 ± 2.6 | 20.2 ± 9.8 | 0.2684 |
| CREA | | 0.05-0.65 mg/dL | 0.5 ± 0.1 | 0.6 ± 0.3 | 0.3412 |
| PHOS | | 5.8-11.1 mg/dL | 7.5 ± 0.6 | 6.1 ± 2.2 | 0.6188 |
| CA | | 5.3-11.6 mg/dL | 10.3 ± 0.2 | 10.4 ± 0.3 | 0.8449 |
| NA | | 135-146 mmol/L | 137.8 ± 1 | 139.5 ± 0.8 | 0.0158 |
| K | | 4.0-5.9 mmol/L | 4.6 ± 0.5 | 4.1 ± 0.4 | 0.2928 |
| CL | | 96-107 mmol/L | 100.5 ± 2.1 | 102.0 ± 2.1 | 0.3197 |
| CO2 | | 17.7-25.7 mmol/L | 25.3 ± 0.8 | 25.3 ± 1.9 | >0.9999 |
| AGAP | | 13.3 -21.3 mmol/L | 16.5 ± 1.1 | 16.3 ± 3.1 | 0.9849 |
| TP | | 5.3-6.9 g/dL | 6.1 ± 0.2 | 6.2 ± 0.3 | 0.5405 |
| ALB | | 2.9-4.8 g/dL | 3.4 ± 0.2 | 3.4 ± 0.1 | 0.9740 |
| GLOB | | 1.8-3.0 g/dL | 2.7 ± 0.1 | 2.8 ± 0.2 | 0.1870 |
| A/G | | N/A | 1.3 ± 0.1 | 1.2 ± 0.0 | 0.5501 |
| ALT | | 20-61 IU/L | 40.2 ± 6.1 | 44.8 ± 18.1 | 0.7362 |
| ALKP | | 16-302 IU/L | 221.7 ± 28.4 | 180.2 ± 61.5 | 0.2119 |
| GGT | | 2-3 IU/L | LOD | LOD | N/A |
| TBIL | | N/A mg/dL | LOD | LOD | N/A |
| CHOL | | 40-281 mg/dL | 97.3 ± 3 | 110.2 ± 15.3 | 0.1055 |
| AMY | | 326-2246 IU/L | 2193.8 ± 758.4 | 1900.2 ± 571.8 | 0.6799 |
| LIPA | | 10-150 IU/L | 104.7 ± 17.5 | 96.3 ± 23.1 | 0.7825 |

mg/dL: milligrams per deciliter; g/dL: grams per deciliter; mmol/L: millimoles per liter; IU/L: units per liter; LOD = values below the limit of detection for this assay.

Data presented as mean ± standard deviation.

**^†^**P values were determined using a Šídák's multiple comparisons test against the means of vehicle day 1 and glyoxylate treated day 1 rats.

**Supplementary Table 7. Comprehensive metabolic panel results in rats 5 days after treatment with 60 mg/kg IP glyoxylate.**

| **Analyte** | **Reference Range** | | **Vehicle Control (n=6)** | **Glyoxylate (n=6)** | ***P* value^†^** |
| --- | --- | --- | --- | --- | --- |
| GLU | | 50-135 mg/dL | 160.8 ± 21.5 | 184.3 ± 29.8 | 0.4587 |
| BUN | | 13-28 mg/dL | 15.0 ± 3.2 | 21.3 ± 4.3 | 0.1330 |
| CREA | | 0.05-0.65 mg/dL | 0.4 ± 0.1 | 0.5 ± 0.1 | 0.8519 |
| PHOS | | 5.8-11.1 mg/dL | 6.5 ± 1.4 | 5.9 ± 1.5 | 0.7814 |
| CA | | 5.3-11.6 mg/dL | 10.6 ± 0.2 | 10.5 ± 0.2 | 0.3030 |
| NA | | 135-146 mmol/L | 139.3 ± 1 | 140.5 ± 1.0 | 0.1017 |
| K | | 4.0-5.9 mmol/L | 4.2 ± 0.5 | 4.0 ± 0.3 | 0.6800 |
| CL | | 96-107 mmol/L | 101.5 ± 1.4 | 103.3 ± 1.8 | 0.1923 |
| CO2 | | 17.7-25.7 mmol/L | 27.3 ± 0.5 | 23.8 ± 1.6 | 0.0004 |
| AGAP | | 13.3 -21.3 mmol/L | 14.7 ± 1.4 | 17.4 ± 2.5 | 0.1019 |
| TP | | 5.3-6.9 g/dL | 6.2 ± 0.3 | 6.2 ± 0.1 | 0.9144 |
| ALB | | 2.9-4.8 g/dL | 3.4 ± 0.1 | 3.5 ± 0.1 | 0.9003 |
| GLOB | | 1.8-3.0 g/dL | 2.8 ± 0.1 | 2.8 ± 0.1 | 0.8599 |
| A/G | | N/A | 1.3 ± 0.1 | 1.2 ± 0.1 | 0.5501 |
| ALT | | 20-61 IU/L | 40.3 ± 7.1 | 49.2 ± 10.1 | 0.3512 |
| ALKP | | 16-302 IU/L | 214.8 ± 37.7 | 186.0 ± 38.5 | 0.4554 |
| GGT | | 2-3 IU/L | LOD | LOD | N/A |
| TBIL | | N/A mg/dL | LOD | LOD | N/A |
| CHOL | | 40-281 mg/dL | 104.3 ± 7.7 | 102.3 ± 13.1 | 0.9391 |
| AMY | | 326-2246 IU/L | 2254.3 ± 675.7 | 2025.3 ± 395.5 | 0.7709 |
| LIPA | | 10-150 IU/L | 107.5 ± 21.8 | 108.5 ± 27.7 | 0.9964 |

mg/dL: milligrams per deciliter; g/dL: grams per deciliter; mmol/L: millimoles per liter; IU/L: units per liter; LOD = values below the limit of detection for this assay.

Data presented as mean ± standard deviation.

**^†^**P values were determined using a Šídák's multiple comparisons test against the means of vehicle day 5 and glyoxylate treated day 5 rats.

**SUPPLEMENTARY REFFERENCES**

Dunn WB, Broadhurst D, Begley P, Zelena E, Francis-McIntyre S, Anderson N, Brown M, Knowles JD, Halsall A, Haselden JN et al. 2011. Procedures for large-scale metabolic profiling of serum and plasma using gas chromatography and liquid chromatography coupled to mass spectrometry. Nat Protoc. 6(7):1060-1083.
